# Pathogenic *Leptospira* are widespread in the urban wildlife of southern California

**DOI:** 10.1038/s41598-023-40322-2

**Published:** 2023-09-01

**Authors:** Sarah K. Helman, Amanda F. N. Tokuyama, Riley O. Mummah, Nathan E. Stone, Mason W. Gamble, Celine E. Snedden, Benny Borremans, Ana C. R. Gomez, Caitlin Cox, Julianne Nussbaum, Isobel Tweedt, David A. Haake, Renee L. Galloway, Javier Monzón, Seth P. D. Riley, Jeff A. Sikich, Justin Brown, Anthony Friscia, Jason W. Sahl, David M. Wagner, Jessica W. Lynch, Katherine C. Prager, James O. Lloyd-Smith

**Affiliations:** 1 Department of Ecology and Evolutionary Biology, University of California, Los Angeles, CA USA; 2 Institute of the Environment and Sustainability, University of California, Los Angeles, CA USA; 3 The Pathogen and Microbiome Institute, Northern Arizona University, Flagstaff, AZ USA; 4 Evolutionary Ecology Group, Department of Biology, University of Antwerp, Antwerp, Belgium; 5 Wildlife Health Ecology Research Organization, San Diego, CA USA; 6 Veterans Affairs Greater Los Angeles Healthcare System, Los Angeles, CA USA; 7 The David Geffen School of Medicine, University of California, Los Angeles, CA USA; 8 Centers for Disease Control and Prevention, Atlanta, GA USA; 9 Natural Science Division, Pepperdine University, Malibu, CA USA; 10 Santa Monica Mountains National Recreation Area, National Park Service, Thousand Oaks, CA USA; 11 Department of Integrative Biology and Physiology, University of California, Los Angeles, CA USA; 12 Institute for Society and Genetics, University of California, Los Angeles, CA USA

**Keywords:** Ecology, Urban ecology, Ecological epidemiology

## Abstract

Leptospirosis, the most widespread zoonotic disease in the world, is broadly understudied in multi-host wildlife systems. Knowledge gaps regarding *Leptospira* circulation in wildlife, particularly in densely populated areas, contribute to frequent misdiagnoses in humans and domestic animals. We assessed *Leptospira* prevalence levels and risk factors in five target wildlife species across the greater Los Angeles region: striped skunks (*Mephitis mephitis*), raccoons (*Procyon lotor*), coyotes (*Canis latrans*), Virginia opossums (*Didelphis virginiana*), and fox squirrels (*Sciurus niger*). We sampled more than 960 individual animals, including over 700 from target species in the greater Los Angeles region, and an additional 266 sampled opportunistically from other California regions and species. In the five target species seroprevalences ranged from 5 to 60%, and infection prevalences ranged from 0.8 to 15.2% in all except fox squirrels (0%). *Leptospira* phylogenomics and patterns of serologic reactivity suggest that mainland terrestrial wildlife, particularly mesocarnivores, could be the source of repeated observed introductions of *Leptospira* into local marine and island ecosystems. Overall, we found evidence of widespread *Leptospira* exposure in wildlife across Los Angeles and surrounding regions. This indicates exposure risk for humans and domestic animals and highlights that this pathogen can circulate endemically in many wildlife species even in densely populated urban areas.

## Introduction

Leptospirosis, the disease caused by pathogenic bacteria from the genus *Leptospira*, is reported to be the most widespread zoonotic disease in the world^1–3^. *Leptospira* spp. are generalist pathogens capable of infecting a broad range of primarily mammalian hosts^4,5^. They are considered emerging pathogens of concern^6^, with one million human cases and 60,000 deaths estimated globally each year^7,8^, and many more cases likely undiagnosed^9^. Given the extensive global impact of *Leptospira* spp., it is surprising that their ecology and epidemiology remain understudied^10^. Though many pathogens, including all zoonoses, are capable of infecting multiple hosts, studying complex generalist pathogen systems remains a challenge, particularly in wildlife for which extensive, multi-host surveillance is required but resources and sample access are often limited^11,12^. Studying *Leptospira* poses additional challenges since host–pathogen epidemiology differs widely across host species and strains, and multiple *Leptospira* strains can co-circulate in communities. Consequently, extensive knowledge gaps remain regarding the role many hosts play in *Leptospira* transmission and the risks they pose to other host species, including humans. Epidemiological patterns also vary across climates and locations, with greater attention paid to the high incidence of human leptospirosis observed in wet tropical regions, and less concern historically placed on urban transmission, especially in more developed countries^1,8^. These complexities underscore the importance of broad surveillance efforts to inform veterinary and public health efforts, particularly in densely populated urban settings where the potential for human-wildlife contact is high and *Leptospira* transmission dynamics remain poorly understood.

Awareness of *Leptospira* genomic diversity has increased in recent years, with pathogenic *Leptospira* bacteria now categorized into 40 species^9,13,14^. Pathogenic *Leptospira* strains are classified into over 250 serovars based on cell surface antigen similarities and serologic reactivity^4^, with related serovars traditionally grouped into serogroups, though neither serovar nor serogroup are reliable predictors of *Leptospira* species^15^. Transmission usually occurs after leptospires are shed in the urine of infected hosts, leading to environmental contamination that can indirectly infect susceptible hosts via mucous membranes or abraded skin^16–19^. Following this contact, leptospires colonize the kidneys and other organs, which can cause pathology and associated clinical signs ranging from mild flu-like symptoms to fulminant multi-organ system failure and death^17,20^. Disease severity varies across *Leptospira* serovars and host species, with some broad host-serovar associations. Historically, the literature has categorized *Leptospira* epidemiology into: (i) maintenance hosts (and host-adapted serovars) that are likely to exhibit asymptomatic and/or chronic infections and maintain circulation of the pathogen, or (ii) accidental hosts (and non-host-adapted serovars) that exhibit symptomatic infections and do not maintain pathogen circulation^1^. These dichotomous classifications have been challenged in recent years, and there is clear evidence that clinical manifestations range widely irrespective of population-level circulation of the pathogen^6,21,22^. Evidence of infection is typically determined from urine or kidney samples using either polymerase chain reaction (PCR) to detect *Leptospira* DNA or culturing to detect live infectious leptospires. The most widely used diagnostic test for *Leptospira* spp. is the microscopic agglutination test (MAT)^4,23^, which tests serum for anti-*Leptospira* antibodies to assess past exposure. Serum MAT panels typically include serovars known or suspected to circulate in the geographic region of interest.

Despite increasing reports of *Leptospira* in urban areas, including dramatic outbreaks linked to flooding^24^, significant knowledge gaps remain regarding the prevalence and transmission risk across a variety of potential hosts in urban environments. Urbanization is increasing at a global scale^25,26^ and can influence wildlife pathogen dynamics through many mechanisms (e.g. altered community structure and contact rates^27^), with high densities of humans and domestic animals providing increased opportunity for cross-species contacts and possible spillover of infection^28^. Unhoused individuals in crowded urban areas may be at increased risk for zoonotic disease transmission due to poor sanitation and intensified contact with urban wildlife species, including in high-income countries where urban homelessness is a growing public health crisis^29^. Since changes to transmission risk are pathogen- and host-specific, investigations into pathogen dynamics and host diversity are critical for urban disease management and risk assessment. This is especially true for *Leptospira*, as most studies in cities have focused on rodents in high-density urban centers^7,30–33^, with much less attention placed on other urban-adapted wildlife that may influence the ecology of this important zoonosis^1,34,35^.

In the United States, many *Leptospira* infections in humans, domestic dogs, horses, and livestock are associated with spillover from wildlife^36–38^. Increasing reports of leptospirosis in domestic dogs^39,40^ and humans in some locations (e.g. Hawaii and California^41–43^) further highlight the need for expanded surveillance in wildlife. One recent study concluded that *Leptospira* exposure is common in a variety of wildlife across the country, and called for more studies investigating the relationship between serology and shedding to better understand the risk this poses to the health of humans, domestic pets, and livestock^44^. In California, a wildlife survey in the 1970s reported *Leptospira* exposure or active infections in multiple species, including coyotes (*Canis latrans*), raccoons (*Procyon lotor*), and striped skunks (*Mephitis mephitis*)^45^, and other surveys have detected *Leptospira* antibodies in black bears (*Ursus americanus*)^46^ and feral pigs (*Sus scrofa*)^47^. A recent survey in northern California reported *Leptospira* in multiple mesocarnivore and rodent species^35^, with both skunks and raccoons identified as potential reservoir hosts for *Leptospira interrogans* serovar Pomona^48^ – a serovar with a long history of circulation in terrestrial mammals on the California Channel Islands and California sea lions (*Zalophus californianus*) along the California coast^21,22,49–53^.

In contrast with northern and coastal regions, there has been little surveillance for *Leptospira* in inland southern California. The greater Los Angeles area features a range of landscapes, from natural and agricultural land to a dense urban center, as well as many wildlife species that are potential *Leptospira* carriers. There are scattered reports of *Leptospira* exposure in some local animals, including dogs^54,55^, deer (*Odocoileus hemionus*)^56^, mountain lions (*Puma concolor*) and bobcats (*Lynx rufus*)^57^, but prior studies have had limited host, geographic, and temporal ranges. The paucity of region-specific surveillance data in greater Los Angeles, the second largest metropolis in the United States^58^, represents a critical public health gap, and more comprehensive wildlife screening is needed to assess zoonotic spillover risk from the full range of susceptible wildlife hosts.

We conducted the first in-depth surveillance of *Leptospira interrogans* in wildlife in the greater Los Angeles area to shed light on the prevalence and circulation of this multi-host pathogen across a densely populated and complex urban landscape. We aimed to assess *Leptospira* exposure and active infections in common wildlife, harnessing novel tools for phylogenomic analysis of infecting *Leptospira* strains to additionally explore potential transmission links among host species. Serologic reactivity profiles across species provided additional insights into multi-host *Leptospira* ecology, and facilitated additional explorations into predictors of exposure and infections in local wildlife. Improved knowledge of *Leptospira* epidemiology in a greater range of susceptible urban host species, and the diversity of *Leptospira* strains they carry, will provide valuable context for wildlife management agencies and clinicians assessing illness in humans and domestic animals across the region.

## Methods

### Study animals

**Figure 1:**
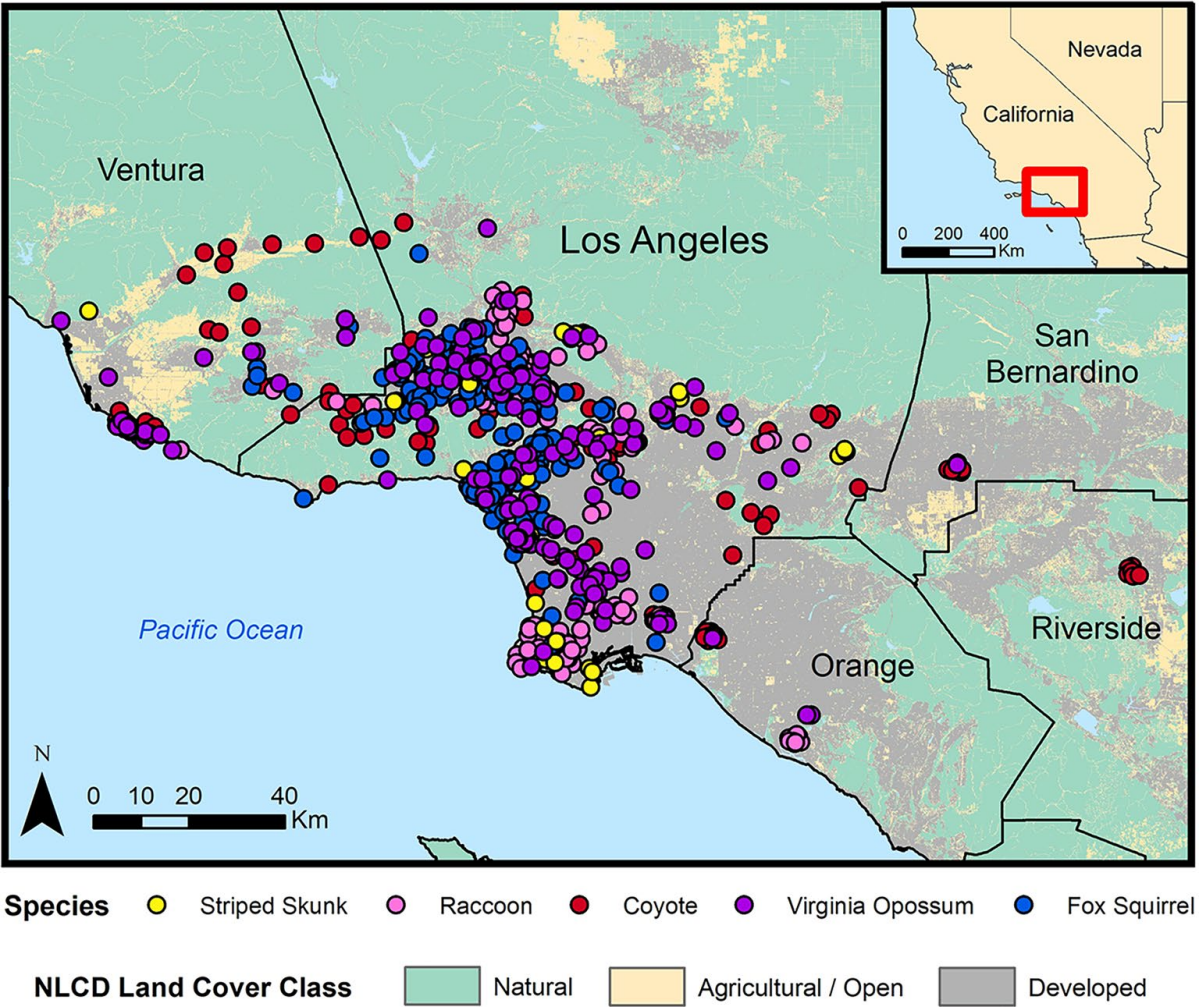
Distribution of *Leptospira* sample locations for the five target wildlife species in the greater Los Angeles region (2015–2020). Land cover data were obtained from the National Land Cover Database (2019).

No live animals were captured or handled explicitly for the purposes of this study, and all wildlife sampling was carried out in accordance with relevant guidelines and regulations. This study focused primarily on wildlife from the greater Los Angeles region in southern California, with most results coming from opportunistically collected carcasses. Opportunistic carcass and sample collection was approved by the California Department of Fish and Wildlife (scientific collecting permits SC-13267 and SC-13700) and took place from September 2015 to June 2020. A small number of mountain lion (n = 11) and coyote (n = 19) samples were previously collected as part of National Park Service mountain lion and coyote studies, with capture and handling procedures permitted through the California Department of Fish and Wildlife (scientific collection permit SC-0005636) and the National Park Service Institutional Animal Care and Use Committee. Three additional serum samples were included from live-trapped striped skunks, with capture and handling procedures permitted for T. Stankowich through the California Department of Fish and Wildlife (scientific collection permit SC-006837) and the California State University Long Beach Institutional Animal Care and Use Committee (protocol #334). For the purposes of this study, the greater Los Angeles region refers to Los Angeles County and neighboring counties: Orange, Riverside, San Bernardino, and Ventura. Sample collection focused on our ‘target species’, five common mammals in the Los Angeles region: striped skunks, raccoons, coyotes, Virginia opossums (*Didelphis virginiana*) and fox squirrels (*Sciurus niger*; Table 1, Fig. 1). Collaborating wildlife agencies donated carcasses or existing samples, with the majority coming from animals killed by vehicle collisions or planned wildlife removal, or animals euthanized by animal control or rehabilitation agencies due to illness or injury. Carcasses were necropsied immediately or frozen at -20ºC and thawed in a refrigerator prior to necropsy. Animal measurements and demographic information were collected at the time of necropsy, with age class (adult or juvenile) determined using a combination of animal size and tooth wear^59^.

**Table 1:** Descriptive characteristics for our five target wildlife species.

	Coyote (N = 137)	Fox Squirrel (N = 187)	Raccoon (N = 172)	Striped Skunk (N = 40)	Virginia Opossum (N = 171)	Total (N = 707)
*Age class*						
Adult	100 (73.0%)	154 (82.4%)	136 (79.1%)	22 (55.0%)	133 (77.8%)	545 (77.1%)
Juvenile	32 (23.4%)	26 (13.9%)	32 (18.6%)	13 (32.5%)	27 (15.8%)	130 (18.4%)
Unknown	5 (3.6%)	7 (3.7%)	4 (2.3%)	5 (12.5%)	11 (6.4%)	32 (4.5%)
**Sex**						
Female	63 (46.0%)	84 (44.9%%)	83 (48.3%)	16 (40.0%)	87 (50.9%)	333 (47.1%)
Male	67 (48.9%)	79 (42.2%)	80 (46.5%)	16 (40.0%)	71 (41.5%)	313 (44.3%)
Unknown	7 (5.1%)	24 (12.8%)	9 (5.2%)	8 (20.0%)	13 (7.6%)	61 (8.6%)
*County*						
Los Angeles	82 (59.9%)	182 (97.3%)	152 (88.4%)	38 (95.0%)	147 (86.0%)	601 (85.0%)
Orange	15 (10.9%)	0 (0%)	9 (5.2%)	0 (0%)	3 (1.8%)	27 (3.8%)
Riverside	8 (5.8%)	0 (0%)	0 (0%)	0 (0%)	0 (0%)	8 (1.1%)
San Bernardino	10 (7.3%)	0 (0%)	0 (0%)	0 (0%)	1 (0.6%)	11 (1.6%)
Ventura	22 (16.1%)	5 (2.7%)	11 (6.4%)	2 (5.0%)	20 (11.7%)	60 (8.5%)
*Season*						
Dry	60 (43.8%)	81 (43.3%)	53 (30.8%)	27 (67.5%)	73 (42.7%)	294 (41.6%)
Wet	77 (56.2%)	106 (56.7%)	119 (69.2%)	13 (32.5%)	98 (57.3%)	413 (58.4%)

Within-group percentages are proportions of column totals.

#### Additional species and regions

On an opportunistic basis, we also tested samples provided by other agencies that included non-target regions of California and non-target species, including ground squirrels (*Otospermophilus beecheyi*), desert cottontails (*Sylvilagus audubonii*), feral pigs, bobcats, mountain lions, gray foxes (*Urocyon cinereoargenteus*), and red foxes (*Vulpes vulpes*). A total of 266 additional samples were collected from 11 species. This included non-target species in Los Angeles County (n = 74) and Ventura County (n = 24), and all species from the following additional counties: Napa (n = 15), Monterey (n = 86), San Luis Obispo (n = 61) and Santa Barbara (n = 6; Supplementary Table S1).

### Sample collection

Serum and urine samples collected at external agencies were analyzed and included when available. From fresh carcasses processed at UCLA, intracardiac blood was collected into serum separation tubes, then kept in a cooler with an ice pack until centrifugation (1350 × g for 10–15 min). Kidney samples were collected from all necropsies, and urine was collected when available using cystocentesis. The largest possible kidney sample that would fit in a 58 ml Whirl–PakⓇ was excised and homogenized in the sealed Whirl–PakⓇ using manual pressure (approximate size: entire kidney from smaller mammals such as squirrels or half a kidney from larger mammals such as coyotes). Serum and urine samples were transferred into cryovials prior to storage, and all cryovials and Whirl-PaksⓇ were stored at − 20 ºC or − 80 ºC prior to testing (− 80 ºC preferred and utilized when possible).

### Leptospira PCR analysis

The presence of pathogenic *Leptospira* DNA was determined in urine and homogenized kidney samples. For all species except mountain lions, samples collected from 2015 to 2017 were analyzed at the Hollings Marine Laboratory (Charleston, South Carolina, USA) using a quantitative polymerase chain reaction (qPCR) assay targeting the *lipL32* gene as detailed in Wu et al.^60^. Samples collected after 2017 were analyzed at Colorado State University Veterinary Diagnostic Laboratory (Denver, Colorado, USA) using the VetMAX™ qPCR Master Mix kit and the primers specified by Wu et al.^60^. Minor modifications to the protocol resulted in slightly higher sensitivity in the later samples, but results across laboratories were broadly consistent^61^. Samples that had a cycle threshold value less than 37 were considered PCR-positive and evidence of active infections. Samples from mountain lions were analyzed at the California Animal Health and Food Safety Laboratory (Davis, California, USA) using their standard protocol^62^.

### Genetic sequencing and phylogenetic analyses

#### DNA capture and enrichment methods

To facilitate robust genomic level species identification and phylogenomic analyses, four *Leptospira-*positive DNA samples (CM-61, MM-3, PL-20, and PL-117) were subjected to pan pathogenic *Leptospira* DNA capture and enrichment as described in detail elsewhere^63^. Briefly, the sample DNAs were diluted separately to ~ 4 ng/µL in a volume of 40µL, sonicated to an average size of 225 bp using a Q800R2 sonicator (QSonica, Newtown, CT, USA), and then short-read next-generation libraries were prepared using Agilent Sure-Select methodology. The libraries were then subjected to one (MM-3 and PL-20) or two (CM-61 and PL-117) rounds of DNA capture and enrichment and then sequenced on an Illumina MiSeq instrument using a MiSeq v3 600 cycle kit (2 × 300 bp reads).

#### Read classifications

To estimate the percentage of *Leptospira* reads in the enriched sequences, reads were mapped against the standard Kraken database with Kraken v2.1.2^64^.

#### Read mapping and phylogenomics

Single nucleotide polymorphisms (SNPs) were identified among the four enriched genomes and > 340 publicly available *L. interrogans* genomes (GenBank accession numbers provided in Supplementary Fig. S1 and Supplementary Fig. S2) by aligning reads against reference genome *L. interrogans* serovar Copenhageni strain Fiocruz L1-130 (GCA_000007685.1) using minimap2 v2.22^65^ and calling SNPs from the BAM file with GATK v4.2.2^66^ using a depth of coverage ≥ 3 × and a read proportion of 0.9. SNPs that fell within duplicated regions, based on a reference self-alignment with MUMmer v3.1^67^, were filtered from downstream analyses. All of these methods were wrapped by NASP v1.2.1^68^. Maximum likelihood phylogenies were then inferred on the concatenated SNP alignments using IQ-TREE v2.2.0.3 with the “-fast” option, default parameters^69^, and the integrated ModelFinder method^70^; the phylogenies were midpoint rooted. To determine breadth of coverage across the reference, reads were aligned against the reference genome with minimap2 and the per base depth of coverage was calculated with Samtools v1.6^71^.

#### Pomona reference phylogeny and WG-FAST placement of enriched samples

We then utilized the Whole Genome Focused Array SNP Typing (*WG-FAST*) tool, a publicly available pipeline designed for phylogenetically typing samples with only partial SNP profiles. Reads were simulated from genome assemblies with ART vMountRainier^72^ using the command “-p -na -ss MSv3 -l 250 -f 75 -m 300 -s 30”. Based on the position of the enriched reads in a complete *L. interrogans* phylogeny, simulated reads were aligned against *L. interrogans* serovar Pomona str. Pomona (GCA_000216355.3) with minimap2 v2.24^65^ and SNPs were called with GATK v4.2.6.1^66^. A midpoint rooted maximum-likelihood phylogeny was inferred from a concatenated SNP alignment (529 positions out of a core genome size of 4,125,494 nts) with RAxML-NG v. 1.1.0^73^. Enriched reads were inserted into this reference phylogeny with *WG-FAST* v1.2^74^ using default settings.

### Leptospira serology

Past exposure to *Leptospira* was assessed using serum microscopic agglutination testing (MAT). In this test, dark-field microscopy is used to assess the presence of anti-*Leptospira* antibodies in serum by evaluating agglutination (i.e. clumping) when samples are combined with live cultures of *Leptospira* species^23^. Serum samples are tested at doubling dilutions, beginning at 1:100 and continuing until endpoint, with the reported endpoint titers representing the highest dilution that achieved a 50% agglutination using the reference strain being tested. An antibody titer of 1:100 or higher to any serovar was considered positive for *Leptospira* exposure. Samples from 2018 to 2020 were analyzed at the California Animal Health and Food Safety Laboratory (CAHFS; Davis, California, USA) against a panel of 6 serovars that are common in the United States^44,75^: Bratislava, Canicola, Grippotyphosa, Hardjo, Icterohaemorrhagiae, and Pomona (Supplementary Table S2). The CAHFS lab uses *L. interrogans* serovar Copenhageni strain M20 as a representative member of the Icterohaemorrhagiae serogroup, and reports results as Icterohaemorrhagiae; it will henceforth be referred to as Icterohaemorrhagiae since they are used interchangeably on MAT testing^76^. Samples from 2015 to 2017 were analyzed at the Centers for Disease Control and Prevention (CDC; Atlanta, Georgia, USA) using an expanded panel of 20 serovars: Alexi, Australis, Autumnalis, Ballum, Bataviae, Borinca, Bratislava, Canicola, Celledoni, Cynopteri, Djasiman, Georgia, Grippotyphosa, Icterohaemorrhagiae, Javanica, Mankarso, Pomona, Pyrogenes, Tarassovi, and Wolffi (Supplementary Table S2). To assess consistency between the two laboratories, we compared the subset of wildlife samples analyzed at both laboratories (n = 469), demonstrating 98.3% agreement in seropositivity and minor quantitative differences between titers. Serovar cross-reactivity patterns were visualized using heatmaps of serovar titers from all seropositive animals using package ‘Heatmap’ in *R* version 4.3.0^77^.

### Analysis of surveillance data

Prevalence was estimated for *Leptospira* exposure and infections. For *Leptospira* serology, individuals could be reactive to multiple serovars, so seroprevalence was calculated for each host species in two ways: proportion positive against any serovar, and proportion positive against each specific serovar. All 95% binomial confidence intervals were estimated using package ‘PropCIs’ in *R*. Additional analyses were done in *R*, and maps were created using ArcGIS version 10.8.2^78^.

Land cover analysis was conducted as detailed in Adducci II et al.^79^ using data from the National Land Cover Database^80^. Sample localities occurred across a diverse landscape gradient, ranging from natural vegetation and open space to the urban core of Los Angeles (Fig. 1). To account for land cover variation within home ranges, we extracted 2019 land cover data (30 m $\times$ 30 m resolution) from home range buffers around each georeferenced sampling locality using the ‘raster’ and ‘rgdal’ packages in *R*. Buffer size varied by species based on home range estimates previously reported in the literature: 5 km^2^ for coyotes^79^, 2 km^2^ for raccoons and striped skunks^81^, 1 km^2^ for opossums^82^, and 0.5 km^2^ for fox squirrels^83^. As detailed in Adducci II et al.^79^, we grouped land cover classifications into three composite categories: urban/suburban (land with 20–100% impervious surface cover), agricultural/open (land used for pastures, crops and open development with < 20% impervious surface cover), and natural (shrubland, forest, grassland and wetland). We then calculated the relative proportions of these three land categories for each individual home range buffer, and used package ‘ggtern’ in *R* to make ternary plots showing *Leptospira* exposure (i.e. presence of antibodies) relative to land categories.

To explore potential predictors of *Leptospira* exposure (as indicated by an antibody titer of 1:100 or higher to any serovar), logistic regressions were conducted with the following covariates considered: age class, sex, season (wet Nov-April vs. dry May-Oct)^35^, and composite land classification (i.e. percentage of the home range that was developed vs. agricultural/open vs. natural). Since the correlation was high between developed and agricultural/open land (Spearman’s $\rho =-0.81$), and developed and natural land (Spearman’s $\rho =-0.90$), the developed category was excluded from these models.

Antibody titers have been identified as significant predictors of *Leptospira* shedding and PCR status (i.e. active infections) in California sea lions^22,84^. We therefore explored this association in raccoons, the only species with a sufficient number of paired antibody-PCR samples available (n = 81). Since these data exhibited complete separation (i.e. a clear distinction between the two outcomes), we applied a Firth’s bias-reduced logistic regression, a penalized maximum likelihood approach that is effective in the presence of data separation^85^, using the ‘logistf’ package in *R* to assess the association between antibody titers and PCR data.

## Results

### Broad patterns of Leptospira exposure and infection

**Figure 2:**
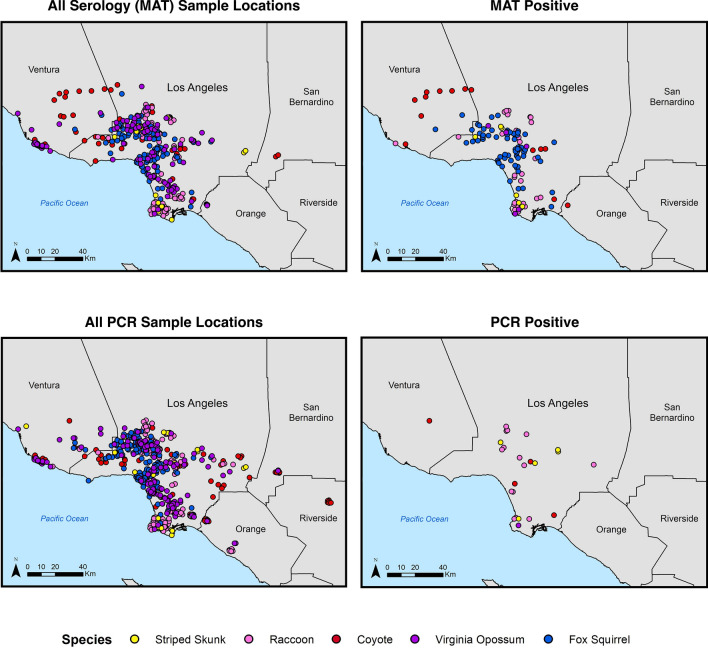
Locations of sample collection and associated *Leptospira* exposure and infection results for the five target wildlife species. Host sample locations are indicated in the left-hand panels, with the locations of animals with positive results shown in maps on the right. Samples tested by MAT for serum antibodies are shown in the top row, and those tested by PCR for pathogenic *Leptospira* DNA are shown on the bottom row.

We detected evidence of *Leptospira* exposure in all five target species sampled in the greater Los Angeles region (Fig. 2, Table 2). Overall seroprevalence in each species was calculated as the proportion of samples that were positive against any serovar. Fox squirrels had the highest seroprevalence at 60.6% (Table 2), though most maximum titers in this species were low (Table 3). Seroprevalence was moderate in mesocarnivores (32.6% in raccoons, 28.6% in striped skunks, 25.9% in coyotes), and low (5.2%) in opossums.

**Table 2:** *Leptospira* exposure and infection by species.

Common name	Scientific name	*Leptospira* Exposure (MAT)			*Leptospira* Infections (PCR)		
		POS	n	%POS (95% CI)	POS	n	%POS (95% CI)
Striped Skunk	*Mephitis mephitis*	6	21	28.6 (11.3–52.2)	5	33	15.2 (5.1–31.9)
Raccoon	*Procyon lotor*	31	95	32.6 (23.4–43.0)	14	162	8.6 (4.8–14.1)
Coyote	*Canis latrans*	14	54	25.9 (15.0–39.7)	4	108	3.7 (1.0–9.2)
Virginia Opossum	*Didelphis virginiana*	5	97	5.2 (1.7–11.6)	1	131	0.8 (0.0–4.2)
Fox Squirrel	*Sciurus niger*	66	109	60.6 (50.7–69.8)	0	148	0 (0.0–2.5)
Total		122	376	32.4 (27.7–37.4)	24	582	4.1 (2.7–6.1)

*Leptospira* antibody (MAT) and DNA (PCR) results in the five target host species sampled in the greater Los Angeles region. Antibody results include seropositives to all serovars tested.

**Table 3:** Maximum antibody titers for our five target wildlife species.

Common name	Scientific name	Serovar	100	200	400	800	1600	3200	≥ 6400	#Positive / Total	Percentage (95% CI)
Striped Skunk	*Mephitis mephitis*	Pomona	1		1		1		2	5/5	100 (47.8–100)
Raccoon	*Procyon lotor*	Bratislava	2			1				3/30	10 (2.1–26.5)
		Icterohaemorrhagiae	2	1		1				4/30	13.33 (3.8–30.7)
		Pomona	6	5	5	3	2	2	3	26/30	86.67 (69.3–96.2)
Coyote	*Canis latrans*	Bratislava	2							2/7	28.57 (3.7–71)
		Icterohaemorrhagiae		1						1/7	14.29 (0.4–57.9)
		Pomona	1	1	1		1		1	5/7	71.43 (29–96.3)
Virginia Opossum	*Didelphis virginiana*	Icterohaemorrhagiae	1							1/2	50 (1.3–98.7)
		Pomona							1	1/2	50 (1.3–98.7)
Fox Squirrel	*Sciurus niger*	Bratislava	5	12	8	4	2			31/60	51.67 (38.4-64.8)
		Icterohaemorrhagiae	25	11	6	4	1	1		48/60	80 (67.7–89.2)

Maximum antibody titers and corresponding serovars for each individual from the five target species sampled in the greater Los Angeles region, reported for our five primary serovars (Bratislava, Canicola, Grippotyphosa, Icterohaemorrhagiae, and Pomona). Only individuals with a highest titer to one of these five primary serovars are included in the totals. In cases where there were ties for maximum titer, both serovars were counted in the table.

Of the 582 target animals tested by PCR in the greater Los Angeles region, 24 (4%) were PCR positive, with active infections detected in all species except fox squirrels (Table 2, Fig. 2). Infection prevalence ranged from 0.8% in opossums to 15% in skunks, with coyotes and raccoons intermediate at 3.7% and 8.6%, respectively. Infection prevalence was consistently lower than corresponding seroprevalence levels for each species, with no active infections detected in fox squirrels despite seroprevalence being highest in this species (Table 2).

### Phylogenetic typing of Leptospira

We used DNA capture and enrichment to enable sequencing of *Leptospira* DNA present in DNA extracts obtained from kidney or urine samples from four of our PCR-positive animals: one coyote (CM-61), one striped skunk (MM-3), and two raccoons (PL-20 and PL-117). Two samples underwent two rounds of enrichment and had very high proportions of sequencing reads assigned to *Leptospira*: 90.52% for PL-117 (1,615,997 of 1,785,218 total reads) and 84.71% for CM-61 (1,194,246/1,409,734). The other two samples underwent just one round of enrichment and had lower proportions of reads assigned to *Leptospira*: 11.05% for PL-20 (141,648/1,281,984) and 6.98% for MM-3 (115,700/1,658,612).

**Figure 3:**
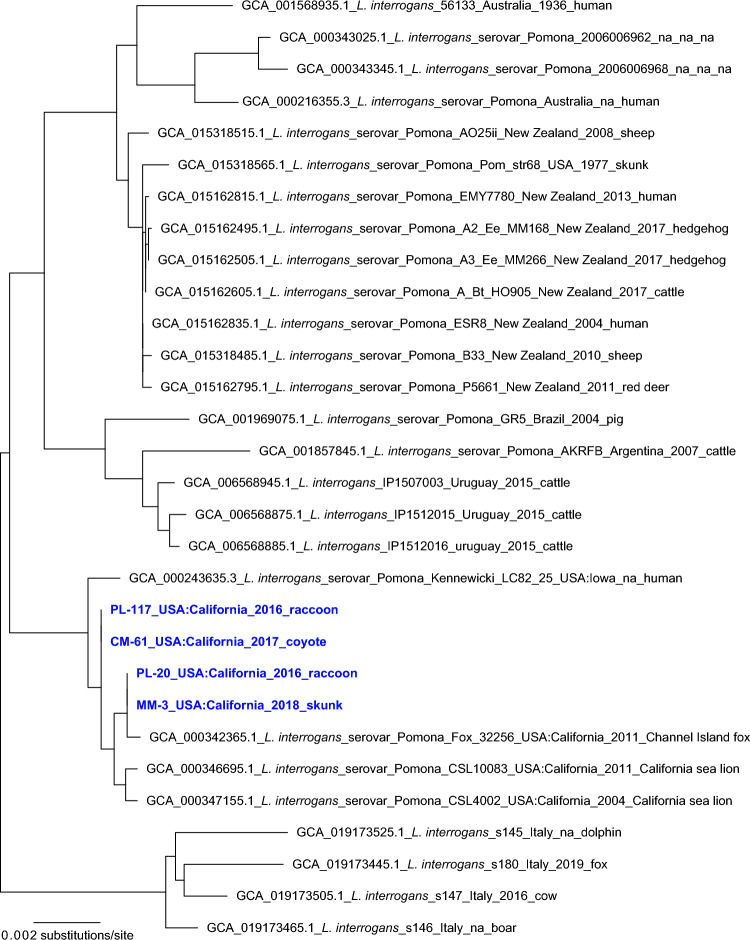
*L. interrogans* serovar Pomona phylogeny. A maximum likelihood phylogeny of 26 *L. interrogans* serovar Pomona genomes together with enriched *Leptospira* genomic DNA from four PCR-positive animals in this study (highlighted with blue text). The tree was inferred from a concatenated SNP alignment (529 positions out of a core genome size of 4,125,494nts) with RAxML-NG v. 1.1.0. *Leptospira* from these four animals cluster in a single clade also containing isolates collected from two California sea lions sampled in 2011 and one Channel Island fox sampled in 2004.

We used several approaches to phylogenetically characterize the *Leptospira* strains infecting these four animals based on the genomic sequence data we generated using targeted DNA capture and enrichment. First, we aligned the sequence reads from these four samples against reference genome *L. interrogans* serovar Copenhageni strain Fiocruz L1-130, which revealed coverages of 82.67% for PL-117 and 84.07% for CM-61, which both underwent two rounds of enrichment, and 53.17% for PL-20 and 54.61% for MM-3, which both underwent just one round of enrichment. We then constructed a phylogeny using the core genome shared by all four samples and the > 340 publicly available *L. interrogans* genomes, which corresponded to just 19.27% (891,916/4,627,366) of the reference genome. Within this phylogeny (Supplementary Fig. S1), the four enriched samples formed a single clade that was embedded within a larger clade containing published sequences from *L. interrogans* serovar Pomona isolates; the three most closely related isolates from outside our current study were collected within the last two decades in coastal California from two California sea lions (CSL4002 and CSL10083) and one Channel Island fox (*Urocyon littoralis*; Fox 32256). We then repeated this approach excluding the less-enriched samples PL-20 and MM-3, which increased the shared core genome among the analyzed samples/isolates to 41.53% (1,921,606/4,627,366) of the reference genome; the phylogenetic patterns remained unchanged (Supplementary Fig. S2). Finally, we used *WG-FAST* to map the *Leptospira* sequence reads from all four samples onto a reference phylogeny developed independently using only the published whole genome sequences from isolates, such that our new enriched genomes did not influence the tree topology. Again, the key patterns were preserved: our four new sequences were placed clearly within *L. interrogans* serovar Pomona and were clustered together along with recent isolates from California sea lions and a Channel Island fox (Fig. 3).

### Serological reactivity patterns

We analyzed patterns of serological reactivity to gain further insight into the *Leptospira* strains circulating in the Los Angeles region. We first investigated the five main serovars against which all samples were tested (serovars Bratislava, Canicola, Grippotyphosa, Icterohaemorrhagiae, and Pomona), and evaluated seroprevalence and titer magnitudes against each serovar (Supplementary Fig. S3, Supplementary Table S3). In skunks, raccoons, and coyotes, the maximum MAT titers in each individual were most frequently against serovar Pomona (100%, 87%, and 71%, respectively; Table 3); among individuals with higher titers (1:1600 or above), the maximum titer was always against serovar Pomona. Maximum MAT titer is an imperfect indicator of the infecting serovar, due to well-known challenges with MAT cross-reactivity^74,86^, but in our study the relationship was supported by the two animals that had both serologic and phylogenomic analyses conducted: the coyote (CM-61) and skunk (MM-3) samples both had highest titers to serovar Pomona (1:102,400 and 1:12,800, respectively), and both were phylogenomically clustered with isolates of *L. interrogans* serovar Pomona (Fig. 3). Fox squirrels exhibited a pattern that was distinct from the other hosts, with highest titers most often to serovar Icterohaemorrhagiae (80%), which was not highly reactive in any of the other host species. Of the two opossums that exhibited reactions against these 5 serovars, one individual (the only opossum in this study with an active infection) had a maximum titer to serovar Pomona (1:12,800).

**Figure 4:**
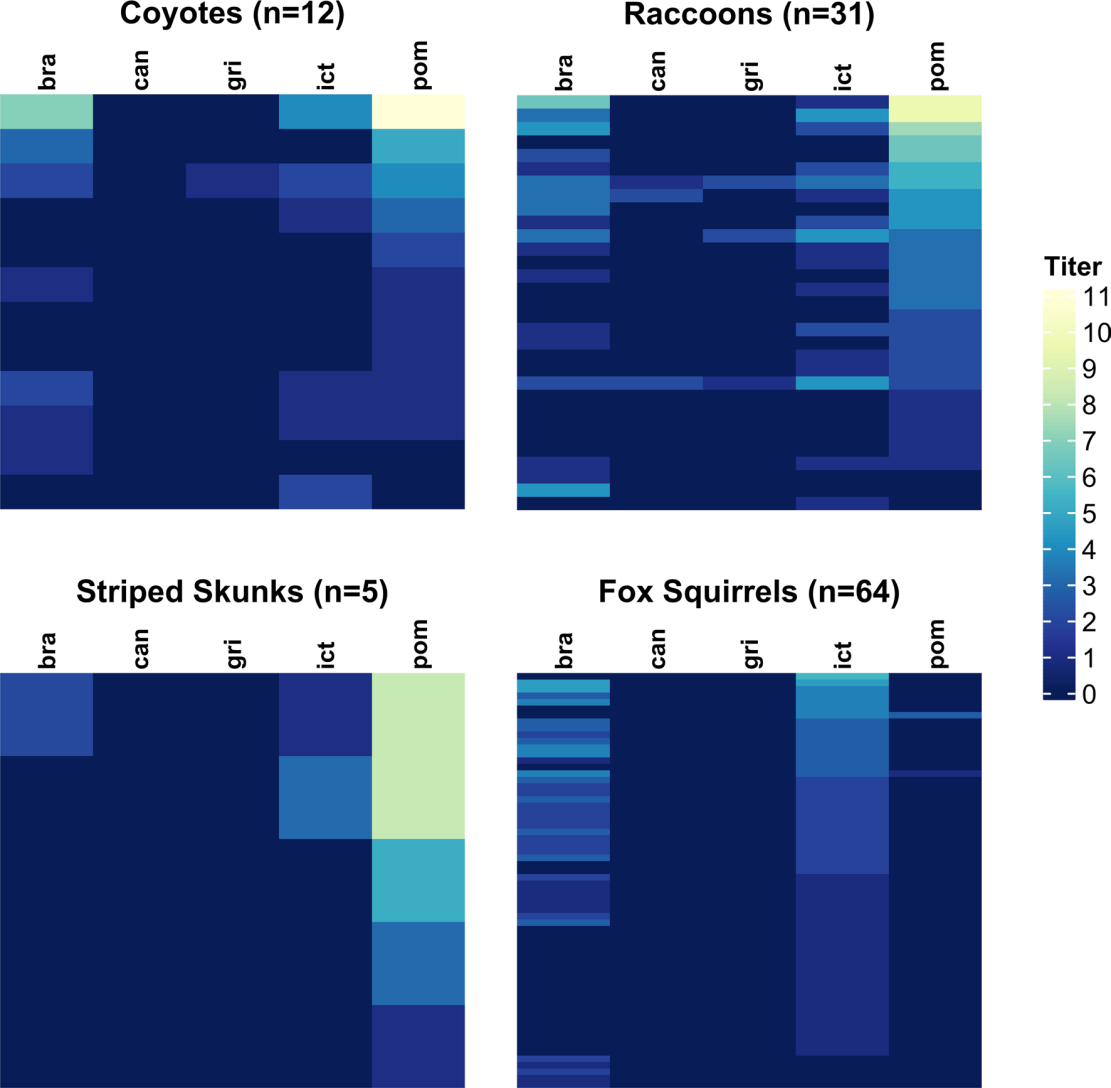
Serovar reactivity profiles for target species in the greater Los Angeles region. Data from all seropositive individuals are shown for the five serovars tested at both laboratories: Bratislava (‘bra’), Canicola (‘can’), Grippotyphosa (‘gri’), Icterohaemorrhagiae (‘ict’), and Pomona (‘pom’). Each row of colored bars represents MAT titers for an individual animal, with rows ordered by titer values for the serovar with the highest maximum titers in each species (serovar Pomona for coyotes, raccoons and skunks; serovar Icterohaemorrhagiae in fox squirrels). The colorbar legend shows antibody titer on a log_2_ scale (1:100 equivalent to 1, 1:200 equivalent to 2, 1:400 equivalent to 3, etc.).

To gain insight into possible sources of reactivity against other serovars, we visualized individual seroreactivity profiles using heatmaps as a way to make sense of the multidimensional data from MAT panels (Fig. 4, Supplementary Fig. S4). The mesocarnivores show strongest reactivity against serovar Pomona. Reactions to other serovars tend to co-occur with strong reactions to serovar Pomona, consistent with the titers against other serovars being cross-reactions to an infection caused by serovar Pomona. A small number of individuals deviate from this pattern (e.g. one raccoon with high titer against serovar Bratislava and low titer against serovar Pomona, possibly indicating an isolated infection with another strain of *Leptospira*). Fox squirrels show strongest reactivity to serovar Icterohaemorrhagiae (Fig. 4, Supplementary Fig. S4), with other reactivities tending to co-occur.

We then considered MAT results for all 21 tested serovars in target species from all regions (Supplementary Table S3). Broad reactivity patterns in non-target regions were similar to patterns seen in greater Los Angeles with serovar Pomona highlighted in mesocarnivores (Supplementary Fig. S5). Serovar Pomona exhibited the highest seroprevalence in raccoons (40.7%), and was one of the highest in skunks (26.9%) and coyotes (18.9%). Mesocarnivore results from the CDC 20-serovar panel showed Pomona as dominant with strong cross-reactions to Autumnalis and Djasiman, with titers against those serovars higher than Pomona in some cases. Across all seropositive coyotes, maximum titers were most commonly observed to serovars Autumnalis (n = 9/22) and Pomona (n = 6/22), with 55% (n = 12/22) and 64% (n = 14/22) of coyotes positive to serovars Autumnalis and Pomona respectively. In coyotes with paired titers (n = 15), Pomona titers were most strongly correlated with titers against serovars Autumnalis (Spearman’s $\rho =0.87$) and Djasiman (Spearman’s $\rho =0.83$). In aggregate, the data from raccoons, coyotes and skunks are consistent with most infections being caused by serovar Pomona. In opossums, the broader dataset revealed several individuals with low titers against serovar Hardjo, making it the most frequently positive serovar (Supplementary Table S3), though Pomona was the highest titer observed in the one opossum with an active infection. Aside from the low-titer reactions against Hardjo that more than doubled the overall seroprevalence of opossums, the majority of our conclusions in target species did not change with consideration of the expanded serovar panel: Icterohaemorrhagiae is the dominant serovar in fox squirrels and Pomona is dominant in the other target species.

### Associations between serology and PCR results

**Figure 5:**
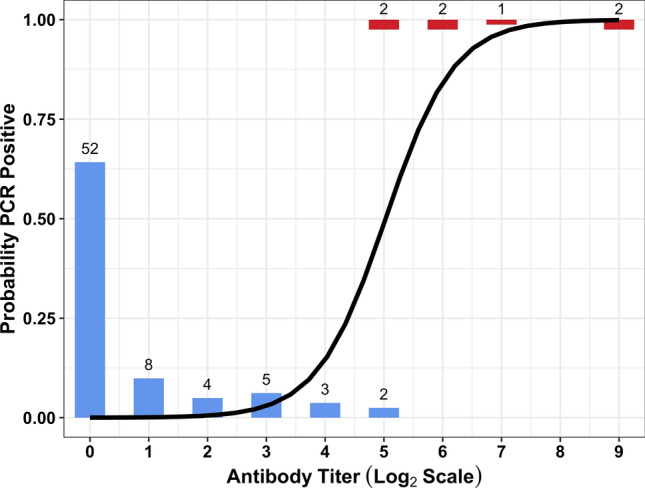
The predicted probability that raccoons are PCR-positive relative to maximum antibody titer to any serovar. The number of individuals with maximum MAT titer at a given level are represented by height of the bars and the corresponding number at the top of the bar. Animals with PCR negative results are shown in blue, and PCR positive individuals are shown in red. Antibody titer (x-axis) is shown on a log_2_ scale (1:100 equivalent to 1, 1:200 equivalent to 2, 1:400 equivalent to 3, etc.). The solid black line shows the best-fitting solution of a Firth’s logistic regression, which predicts that individuals with MAT titers greater than 1:1600 (5 on the log scale) are at least 80% likely to be PCR positive.

To analyze the association between active infections and the maximum antibody titer against any serovar, we used paired PCR and MAT results, for which only raccoons had a sufficient sample size (n = 81). Of those 81 animals, 29 were seropositive, and 86% of seropositive animals (n = 25/29) had maximum titers to serovar Pomona. We found that maximum titer level was a significant predictor of active infection in raccoons (Firth’s logistic regression; p-value = 2.09 × 10^–9^), which aligns with previous studies showing that maximum *Leptospira* MAT titers are effective predictors of infection in California sea lions^22,84^. Individual raccoons with titers above 1:1600 were highly likely to be PCR-positive, and therefore likely to present a transmission risk because of their potential to be actively shedding *Leptospira* (Fig. 5). Intriguingly, among host individuals of other target species that had paired PCR and MAT results, 80% (n = 4/5) of individuals with titers above 1:1600 were PCR-positive (1/1 coyotes, 2/2 skunks, 1/1 opossums and 0/1 fox squirrels).

### Spatial patterns of Leptospira exposure

We detected *Leptospira* exposure (i.e. MAT-positive individuals) throughout the sampled ranges of each host species (Fig. 2). When we evaluated sample location relative to the composite land cover classes, we found indications that different species use the landscape in different ways. For instance, fox squirrel and opossum samples were clustered around areas with higher levels of human development, providing evidence for increased use of urban and suburban regions in these non-native species (Supplementary Fig. S6). In contrast, coyotes and raccoons were found across all land classes. When we evaluated *Leptospira* exposure data in light of these land classes, no clear patterns emerged to distinguish the locations of positive and negative samples (Fig. 2, Supplementary Fig. S6), indicating that *Leptospira* circulates throughout the sampled range of each host species.

To probe how *Leptospira* exposure patterns might be influenced by land cover, season, and demographic factors, we used logistic regressions to assess possible correlates for each species. This was done in all species except skunks, which were excluded due to small overall sample size (n = 23). Of the covariates explored here (age class, sex, season, and land classification), none exhibited significant correlations with *Leptospira* exposure in any of the target species (all p-values > 0.09 in univariate analyses). This lack of correlation can be visualized as a lack of clustering of positive results in ternary plots (Supplementary Fig. S6), supporting that *Leptospira* is distributed throughout the sampled range of these species.

### Additional species and regions

Overall prevalence levels in the non-target dataset were comparable to the target dataset: 4.4% (n = 4/91) of animals were PCR-positive (2 mountain lions, 1 feral pig and 1 bobcat) and antibodies were detected in 30.6% (n = 64/209), with species-specific seroprevalence levels ranging from 0 to 61% (Supplementary Table S1)*.* Feral pigs had overall seroprevalence of 27% (n = 15/55) and were most frequently reactive to serovars Bratislava, Autumnalis, Djasiman, and Pomona (Supplementary Tables S1 and S3). Of the bobcats tested, 46% (n = 5/11) were seropositive (Supplementary Table S1), with titers to Bratislava and Pomona most frequently positive (Supplementary Table S3). Of the non-target species in the greater Los Angeles region, 10% (n = 3/30) of desert cottontails were seropositive, with the three seropositive individuals exhibiting titers against serovars Georgia, Icterohaemorrhagiae, and Pomona, respectively (Supplementary Table S3). Two mountain lions from the greater Los Angeles region were PCR-positive (18.2%; n = 2/11), with both animals showing clinical pathology consistent with leptospirosis (nephritis) upon necropsy.

## Discussion

We conducted a large-scale survey of *Leptospira* in mainland terrestrial mammals in California, focusing on the understudied region of southern California. We had two goals: (1) to identify *Leptospira* prevalence and potential risk factors in the greater Los Angeles region, and (2) to inform our knowledge of broader multi-host circulation in coastal California wildlife. We identified *Leptospira* exposure in all target species (skunks, raccoons, coyotes, opossums, and fox squirrels), and detected active infections in all target species except fox squirrels. Widespread evidence of exposure, which was not correlated with specific landscape factors, highlights that *Leptospira* spp. are endemic and circulating throughout the ranges of these wildlife species in this major metropolitan area. Our findings extend the evidence that *Leptospira* are widespread in many wildlife species throughout California^35,45^ and potentially link transmission in mainland mammals to pathogen circulation in island and marine mammal species.

In our five target species, we detected low to moderate levels of *Leptospira* infection (0–15%) and markedly higher seroprevalence levels (5–60%). Skunks, raccoons and coyotes all exhibited moderate levels of infection (3.7–15.2%), whereas infection prevalence was low in opossums (0.8%) and fox squirrels (0%). Only one opossum was PCR positive, consistent with prior surveillance of opossums elsewhere in California, which have found no^87^ or low *Leptospira* infection prevalence^35^. Fox squirrels had the highest seroprevalence of all target hosts (60%) despite no infections being detected in this species. This could be due to a shorter duration of carriage, longer duration of titer decay (and hence seropositivity), or potentially an alternate route of transmission (e.g. sexual) and associated tissue distribution which could explain the lack of detection in the urinary tract. In addition to the widespread exposure detected in our target species, we detected exposure in all non-target species except gray foxes, and we provide the first evidence that desert cottontails are exposed to *Leptospira* in California.

In our target species, we detected lower infection prevalence and broadly comparable seroprevalence levels to those reported in more northern regions of California^35^ (Supplementary Table S4). This may reflect true regional differences, though comparisons across studies and laboratories should be considered carefully. Notably, the prior study used a PCR cycle threshold of 45 to define positivity^35^, resulting in higher sensitivity and lower specificity (i.e. a lower risk of false negatives, but a higher risk of false positives) than the cycle threshold of 37 used in this study. This could explain the slightly lower infection prevalence in our study. Regional differences in *Leptospira* infection incidence could also arise from environmental differences between northern and southern California, given that wetter environmental conditions facilitate the bacteria’s survival and transmission. For example, higher rainfall has been associated with higher *Leptospira* incidence in domestic dogs^88^, including in northern California^89^. Expanded analyses with broader sampling ranges and consistent protocols could elucidate possible environmental or seasonal patterns of *Leptospira* in California wildlife*.*

We used two broad approaches to characterize the *Leptospira* strains circulating in our system. For four individual animals (two raccoons, one coyote, and one skunk), we sequenced PCR-positive samples and used phylogenomic analysis to determine that they were infected with *L. interrogans* strains that exhibit little genetic distance from one another, and cluster within clades of *L. interrogans* serovar Pomona. For the broader set of individuals and species in our study, we analyzed patterns of seroreactivity to assess possible circulating strains, though we emphasize that serology cannot conclusively identify the *Leptospira* strain causing an infection^86^. Over half of the seropositive animals in this study were reactive to multiple serovars (67%; n = 124/186). Consideration of individual-level reactivity profiles indicated that most of these are likely cross-reactions since they co-occur with stronger reactions against other serovars (though infection by multiple strains of *Leptospira* is possible, as reported in cattle^15^). Trying to infer the infecting strain from serological data is challenging: this is sometimes assumed to be the serovar with the highest MAT titer, but this approach will fail if the infecting serovar is not on the test panel, or in cases of ‘paradoxical reactions’ where another serovar elicits a higher MAT titer than the true infecting serovar. In this study, coyotes, raccoons, and skunks typically had maximum antibody titers against serovar Pomona, which is consistent with our phylogenomic results, and aligns with findings from northern California where serovar Pomona predominated in mesocarnivores^35^. Conversely, squirrels showed minimal seroreactivity to serovar Pomona and typically had low maximum titers against serovar Icterohaemorrhagiae, which was not highly reactive in other species. In our two individual animals with both serology and genomic data (one raccoon and one coyote), we confirmed that the maximum titer against Pomona was indeed associated with infection by *L. interrogans* serovar Pomona. In a subset of samples tested against a broader array of serovars, some coyotes exhibited highest titers to serovar Autumnalis, though titers against serovar Pomona were also high, and titers against Pomona and Autumnalis were strongly correlated. We note that similar patterns have been reported in other canids (domestic dogs^91^ and Channel Island foxes^61^), including island foxes with culture-confirmed serovar Pomona infections that caused peak titers against serovar Autumnalis^61^. Though further genomic data are needed to identify all distinct strains circulating in Los Angeles wildlife, based on current evidence we propose that there are at least two: a strain of *L. interrogans* serovar Pomona circulating in mesocarnivores (and perhaps other unsampled species), and another strain (with serologic reactivity against serovar Icterohaemorrhagiae) circulating in fox squirrels.

Our finding of widespread *Leptospira* circulation, with clear indications that serovar Pomona may be predominant in multiple host species, has potential implications for ongoing research in marine and terrestrial island mammals in California^21,49–53,61,90^. Phylogenetic analyses of *Leptospira* genomes isolated from California sea lions, northern elephant seals (*Mirounga angustirostris*), Channel Island foxes and island spotted skunks (*Spilogale gracilis amphiala*) show evidence of repeated introductions of new strains of *L. interrogans* serovar Pomona into the broader coastal ecosystem^52^. The source of the introductions to the marine ecosystem is unknown, but mainland terrestrial mammals are one possibility. Our data are consistent with this hypothesis: our phylogenomic analysis of four PCR-positive samples from mainland mesocarnivores showed that they clustered with *L. interrogans* serovar Pomona genomes, and were nested closest to recent *L. interrogans* serovar Pomona isolates from two California sea lions and one Channel Island fox. More genomic data from mainland terrestrial mammals are needed to confirm the timing and direction of transmission links between these mainland and marine ecosystems^91^.

Information on wildlife disease occurrence is crucial to assessing wildlife and human health risks, but collecting samples to support broad-scale wildlife surveillance can be very challenging. New approaches to analyzing existing data and samples can provide valuable tools to scale up and make the most of surveillance efforts. Our work highlights the new insights that can be gained from emerging laboratory techniques like targeted DNA capture and enrichment^63^, which facilitate whole genome-based phylogenomic analyses in the absence of the cultured isolates that *Leptospira* genomics previously required. Novel insights can also be gained from more accessible samples when surveillance efforts are limited. For example, the relationship between *Leptospira* antibody titers and infection status, which has previously not been well characterized in wildlife^44^, may provide a proxy for determining infection prevalence from readily accessible serum samples. We showed that antibody titers are strongly predictive of infection status in raccoons, and similar results in California sea lions^22,84^ indicate a potentially robust general pattern. This could be a useful wildlife screening tool in situations where PCR results are not available or feasible to obtain, but more investigation is needed into the generality of this relationship given the known potential for *Leptospira* to exhibit species- or host-specific patterns. Consequently, using antibody titers as a proxy for infection status may not apply in some host species, such as squirrels, where antibody responses are variable^92,93^, or opossums where some individuals fail to mount an antibody response and seronegative shedding is possible^35,94^.

Baseline knowledge about the prevalence of zoonotic infections in urban wildlife has important utility for public health efforts. Diagnosing early cases of leptospirosis in humans and domestic animals can be challenging due to non-specific clinical signs. Raising clinical awareness about epidemiological risk factors (e.g. the prevalence in sympatric host species) is therefore critical for facilitating accurate diagnostics and disease risk assessments^9^. Broad-scale surveillance efforts also contribute to knowledge of circulating variants, which can be used to rapidly identify or exclude potential sources of transmission. For example, in 2021 there was a leptospirosis outbreak in Los Angeles dogs caused by *L. interrogans* serovar Canicola^55^. Very few individuals in our study showed any reactivity at all against serovar Canicola. This corroborates the conclusion that this outbreak did not originate from local wildlife^55^, highlighting the importance of longitudinal wildlife surveillance in determining (or ruling out) potential sources of outbreaks caused by multi-host pathogens.

This study has some limitations which suggest directions for additional surveillance efforts and future research. Since only five wildlife species could be sampled extensively, there could be unobserved host species contributing to *Leptospira* persistence and transmission in greater Los Angeles. Identifying these cryptic contributors, sometimes referred to as ‘epidemiological dark matter’^12^, remains a frontier in disease ecology and emphasizes the need for ongoing research to understand multi-host pathogen dynamics. We were primarily dependent on collaborating agencies for salvaged and opportunistically collected samples. This led to limited sample sizes with a degree of spatial clustering around collaborator facilities, which potentially reduced our ability to detect spatial patterns. Samples may also have been biased towards more developed areas, with higher road concentrations potentially increasing traffic-related deaths, and higher human densities increasing the likelihood of sick or dead animals being reported. Additionally, the composite land classification used here may have masked finer-scale spatial associations, and our use of circular home range buffers could overlook behavioral patterns in habitat use. Further investigations utilizing other metrics of urbanization (e.g. population density) or individual home ranges and land use patterns could reveal spatial relationships undetected in our analyses.

This study provides the first in-depth look at *Leptospira* ecology in terrestrial wildlife across the greater Los Angeles area. Expanded knowledge of this pathogen in southern California, including comparisons of prevalence levels and serological patterns across host species, provides key insights into multi-host pathogen dynamics and the potential for cross-species transmission, including from wildlife to humans and their pets. Evidence of *Leptospira* circulation in Los Angeles wildlife has been lacking, contributing to the perception that the pathogen does not pose a major risk in the area. Our study found evidence consistent with endemic circulation of at least two distinct strains of *Leptospira* among our five target species, and applied novel *Leptospira* DNA capture and enrichment techniques that yielded preliminary evidence linking mainland mammal transmission to nearby island and marine systems. High levels of exposure and wide geographic distribution indicate that *Leptospira* are ubiquitous across the region, with active infection rates substantial enough to warrant concern and to recommend that domestic dogs in the Los Angeles metropolitan area be vaccinated against this disease. Better understanding of *Leptospira* ecology and transmission dynamics in wildlife is critical to the management of this widely circulating pathogen, highlighting the need for more systematic, broad-scale research efforts to monitor this pathogen in wildlife, domestic animals, and humans.

### Supplementary Information

Supplementary Information.

## Data Availability

Data used for this paper are available at: https://github.com/SarahHelman/Lepto_in_SoCal_urban_wildlife. All sequence data were submitted to the NCBI sequence read archive under accession PRJNA979271. Source code for the Whole Genome Focused Array SNP Typing (*WG-FAST*) pipeline is available at: https://github.com/jasonsahl/wgfast.
