# Supplementary material for: Pathogenic Leptospira are widespread in the urban wildlife of southern California
Source: Sci Rep. 2023 Sep 1;13:14368. doi: 10.1038/s41598-023-40322-2 (PMC10474285; doi:10.1038/s41598-023-40322-2)
Supplement: Supplementary file 1 — Supplementary Information. [file 41598_2023_40322_MOESM1_ESM.pdf]

Table S1: *Leptospira* test results from non-target species and regions.

	Bobcat (N=12)	Coyote (N=20)	Desert Cottontail (N=38)	Feral Pig (N=55)	Gray Fox (N=9)	Ground Squirrel (N=38)	Mountain Lion (N=11)	Raccoon (N=41)	Red Fox (N=5)	Striped Skunk (N=5)	Virginia Opossum (N=32)	Total (N=266)
**MAT**												
Negative	6 (50.0%)	13 (65.0%)	27 (71.1%)	40 (72.7%)	4 (44.4%)	2 (5.3%)	1 (9.1%)	16 (39.0%)	3 (60.0%)	3 (60.0%)	30 (93.8%)	145 (54.5%)
Positive	5 (41.7%)	7 (35.0%)	3 (7.9%)	15 (27.3%)	0 (0%)	2 (5.3%)	1 (9.1%)	25 (61.0%)	2 (40.0%)	2 (40.0%)	2 (6.3%)	64 (24.1%)
Not Tested	1 (8.3%)	1 (5.0%)	8 (21.1%)	0 (0%)	5 (55.6%)	34 (89.5%)	9 (81.8%)	0 (0%)	0 (0%)	0 (0%)	0 (0%)	57 (21.7%)
**PCR**												
Negative	1 (8.3%)	1 (5.0%)	27 (71.1%)	7 (12.7%)	6 (66.7%)	36 (94.7%)	9 (81.8%)	0 (0%)	0 (0%)	0 (0%)	0 (0%)	87 (32.7%)
Positive	1 (8.3%)	0 (0%)	0 (0%)	1 (1.8%)	0 (0%)	0 (0%)	2 (18.2%)	0 (0%)	0 (0%)	0 (0%)	0 (0%)	4 (1.1%)
Not Tested	10 (83.3%)	19 (95.0%)	11 (28.9%)	47 (85.5%)	3 (33.3%)	2 (5.3%)	0 (0%)	41 (100%)	5 (100%)	5 (100%)	32 (100%)	175 (65.8%)
**Age Class**												
Adult	9 (75.0%)	14 (70.0%)	36 (94.7%)	36 (65.5%)	6 (66.7%)	27 (71.1%)	10 (90.9%)	39 (95.1%)	4 (80.0%)	5 (100%)	31 (96.9%)	217 (81.6%)
Juvenile	2 (16.7%)	4 (20.0%)	0 (0%)	19 (34.5%)	2 (22.2%)	11 (28.9%)	1 (9.1%)	2 (4.9%)	1 (20.0%)	0 (0%)	0 (0%)	42 (15.8%)
Unknown	1 (8.3%)	2 (10.0%)	2 (5.3%)	0 (0%)	1 (11.1%)	0 (0%)	0 (0%)	0 (0%)	0 (0%)	0 (0%)	1 (3.1%)	7 (2.6%)
**Sex**												
Female	2 (16.7%)	8 (40.0%)	16 (42.1%)	27 (49.1%)	1 (11.1%)	13 (34.2%)	6 (54.5%)	3 (7.3%)	2 (40.0%)	0 (0%)	7 (21.9%)	85 (32.0%)
Male	6 (50.0%)	9 (45.0%)	10 (26.3%)	26 (47.3%)	5 (55.6%)	24 (63.2%)	5 (45.5%)	7 (17.1%)	2 (40.0%)	3 (60.0%)	15 (46.9%)	112 (42.1%)
Unknown	4 (33.3%)	3 (15.0%)	12 (31.6%)	2 (3.6%)	3 (33.3%)	1 (2.6%)	0 (0%)	31 (75.6%)	1 (20.0%)	2 (40.0%)	10 (31.3%)	69 (25.9%)
**County**												
Los Angeles	2 (16.7%)	0 (0%)	24 (63.2%)	0 (0%)	5 (55.6%)	36 (94.7%)	7 (63.6%)	0 (0%)	0 (0%)	0 (0%)	0 (0%)	74 (27.8%)
Monterey	5 (41.7%)	7 (35.0%)	0 (0%)	0 (0%)	2 (22.2%)	1 (2.6%)	0 (0%)	40 (97.6%)	1 (20.0%)	2 (40.0%)	28 (87.5%)	86 (32.3%)
Napa	0 (0%)	0 (0%)	0 (0%)	15 (27.3%)	0 (0%)	0 (0%)	0 (0%)	0 (0%)	0 (0%)	0 (0%)	0 (0%)	15 (5.6%)
San Luis Obispo	0 (0%)	9 (45.0%)	0 (0%)	40 (72.7%)	0 (0%)	0 (0%)	0 (0%)	1 (2.4%)	4 (80.0%)	3 (60.0%)	4 (12.5%)	61 (22.9%)
Santa Barbara	1 (8.3%)	4 (20.0%)	0 (0%)	0 (0%)	1 (11.1%)	0 (0%)	0 (0%)	0 (0%)	0 (0%)	0 (0%)	0 (0%)	6 (2.3%)
Ventura	4 (33.3%)	0 (0%)	14 (36.8%)	0 (0%)	1 (11.1%)	1 (2.6%)	4 (36.4%)	0 (0%)	0 (0%)	0 (0%)	0 (0%)	24 (9.0%)
**Season**												
Dry	3 (25.0%)	9 (45.0%)	16 (42.1%)	17 (30.9%)	3 (33.3%)	28 (73.7%)	8 (72.7%)	13 (31.7%)	2 (40.0%)	0 (0%)	6 (18.8%)	105 (39.5%)
Wet	9 (75.0%)	10 (50.0%)	22 (57.9%)	29 (52.7%)	6 (66.7%)	10 (26.3%)	3 (27.3%)	28 (68.3%)	3 (60.0%)	5 (100%)	26 (81.3%)	151 (56.8%)
Unknown	0 (0%)	1 (5.0%)	0 (0%)	9 (16.4%)	0 (0%)	0 (0%)	0 (0%)	0 (0%)	0 (0%)	0 (0%)	0 (0%)	10 (3.8%)

Table S2: Table of reference serovars and strains used in microscopic agglutination testing at the United States Centers for Disease Control and Prevention (CDC) and the California Animal Health and Food Safety Lab System (CAHFS).

Laboratory	Species	Serogroup	Serovar	Strain
**CDC**	*L. interrogans*	Australis	Australis	Ballico
	L. interrogans	Australis	Bratislava	Jez-Bratislava
	L. interrogans	Autumnalis	Autumnalis	Akiyami A
	L. borgpetersenii	Ballum	Ballum	Mus 127
	L. interrogans	Bataviae	Bataviae	Van Tienen
	L. interrogans	Canicola	Canicola	Ruebush
	L. weilii	Celledoni	Celledoni	Celledoni
	L. kirschneri	Cynopteri	Cynopteri	3522 C
	L. interrogans	Djasiman	Djasiman	Djasiman
	L. kirschneri	Grippotyphosa	Grippotyphosa	Moskva V
	L. santarosai	Hebdomadis	Borincana	HS 622
	L. interrogans	Icterohaemorrhagiae	Icterohaemorrhagiae	RGA
	L. interrogans	Icterohaemorrhagiae	Mankarso	Mankarso
	L. interrogans	Javanica	Javanica	Veldrat Bataviae 46
	L. santarosai	Mini	Georgia	LT 117
	L. interrogans	Pomona	Pomona	Pomona
	L. interrogans	Pyrogenes	Pyrogenes	Salinem
	L. interrogans	Pyrogenes	Alexi	HS 616
	L. interrogans	Sejroe	Wolffi	3705
	L. borgpetersenii	Tarassovi	Tarassovi	Perepelitsin
**CAHFS**	*L. interrogans*	Australis	Bratislava	Jez-Bratislava
	L. interrogans	Canicola	Canicola	Hond Utrecht IV
	L. interrogans	Grippotyphosa	Grippotyphosa	Andaman
	L. interrogans	Sejroe	Hardjo	Hardjoprajitno
	L. interrogans	Icterohaemorrhagiae	Copenhageni	M20
	L. interrogans	Pomona	Pomona	Pomona

Table S3: *Leptospira* serovars and antibody titer levels for all California wildlife samples. Total number of animals tested by host species and serovar (n), the number of animals that exhibited a maximum titer at each titer level, the total number of animals that tested positive to each serovar (N), and the row-wise seropositivity (% positive) with binomial confidence intervals (95% CI). Serovars with no positive results for a given host species are not shown.

Common Name	*Scientific Name*	n	Serovar	100	200	400	800	1600	3200	>=6400	N	Prevalence (%)
**Bobcat**	*Lynx rufus*	7	Autumnalis	0	0	0	0	0	1	0	1	14.29 (0.4-57.9)
		7	Bataviae	1	0	0	0	0	0	0	1	14.29 (0.4-57.9)
		11	Bratislava	2	1	0	1	0	0	0	4	36.36 (10.9-69.2)
		11	Canicola	0	0	0	0	1	0	0	1	9.09 (0.2-41.3)
		7	Cynopteri	1	0	0	0	0	0	0	1	14.29 (0.4-57.9)
		7	Djasiman	0	0	0	0	0	1	0	1	14.29 (0.4-57.9)
		11	Grippotyphosa	0	0	0	0	1	0	0	1	9.09 (0.2-41.3)
		11	Icterohaemorrhagiae	0	1	0	0	0	1	0	2	18.18 (2.3-51.8)
		7	Mankarso	0	0	0	0	1	0	0	1	14.29 (0.4-57.9)
		11	Pomona	0	2	0	0	0	1	0	3	27.27 (6-61)
		7	Pyrogenes	0	0	0	0	1	0	0	1	14.29 (0.4-57.9)
		7	Wolffi	1	0	0	0	0	0	0	1	14.29 (0.4-57.9)
**Coyote**	*Canis latrans*	46	Australis	0	1	0	0	0	0	0	1	2.17 (0.1-11.5)
		46	Autumnalis	3	4	2	1	0	2	0	12	26.09 (14.3-41.1)
		74	Bratislava	5	3	1	1	0	0	2	12	16.22 (8.7-26.6)
		74	Canicola	0	1	0	0	0	0	0	1	1.35 (0-7.3)
		46	Cynopteri	1	0	0	0	0	0	0	1	2.17 (0.1-11.5)
		46	Djasiman	2	3	1	2	0	2	0	10	21.74 (10.9-36.4)
		74	Grippotyphosa	2	2	1	0	0	0	0	5	6.76 (2.2-15.1)
		38	Hardjo	1	1	0	0	0	0	0	2	5.26 (0.6-17.7)
		74	Icterohaemorrhagiae	4	3	1	1	0	0	1	10	13.51 (6.7-23.5)
		46	Mankarso	1	3	1	1	0	0	0	6	13.04 (4.9-26.3)
		74	Pomona	5	1	3	1	2	0	2	14	18.92 (10.7-29.7)
		46	Tarassovi	1	0	0	0	0	0	0	1	2.17 (0.1-11.5)
**Desert Cottontail**	*Sylvilagus audubonii*	27	Georgia	0	0	0	1	0	0	0	1	3.7 (0.1-19)
		30	Icterohaemorrhagiae	1	0	0	0	0	0	0	1	3.33 (0.1-17.2)
		30	Pomona	0	1	0	0	0	0	0	1	3.33 (0.1-17.2)
**Feral Pigs**	*Sus scrofa*	55	Autumnalis	4	1	3	0	0	0	0	9	16.36 (7.8-28.8)
		55	Bratislava	4	4	2	0	0	0	0	10	18.18 (9.1-30.9)
		55	Canicola	1	0	0	0	0	0	0	1	1.82 (0-9.7)
		55	Cynopteri	1	1	0	0	0	0	0	2	3.64 (0.4-12.5)
		55	Djasiman	3	1	4	0	0	0	0	8	14.55 (6.5-26.7)
		55	Georgia	0	1	0	0	0	0	0	1	1.82 (0-9.7)
		55	Icterohaemorrhagiae	2	0	1	0	0	0	0	3	5.45 (1.1-15.1)
		55	Mankarso	1	0	1	0	0	0	0	2	3.64 (0.4-12.5)
		55	Pomona	2	0	1	0	1	1	2	7	12.73 (5.3-24.5)
		55	Pyrogenes	0	1	0	0	0	0	0	1	1.82 (0-9.7)
		55	Tarassovi	1	0	0	0	0	0	0	1	1.82 (0-9.7)
**Fox Squirrel**	*Sciurus niger*	69	Australis	2	0	0	0	0	0	0	2	2.9 (0.4-10.1)
		69	Autumnalis	2	2	0	0	0	0	0	4	5.8 (1.6-14.2)
		110	Bratislava	9	15	9	4	2	0	0	39	35.45 (26.6-45.1)
		69	Celledoni	1	0	0	0	0	0	0	1	1.45 (0-7.8)
		51	Hardjo	8	16	7	2	1	0	0	34	66.67 (52.1-79.2)
		110	Icterohaemorrhagiae	29	15	9	5	1	1	0	60	54.55 (44.8-64.1)
		69	Javanica	1	0	0	0	0	0	0	1	1.45 (0-7.8)
		69	Mankarso	1	1	1	0	0	0	0	3	4.35 (0.9-12.2)
		110	Pomona	1	0	1	0	0	0	0	2	1.82 (0.2-6.4)
		69	Pyrogenes	2	0	0	0	0	0	0	2	2.9 (0.4-10.1)
**Ground Squirrel**	*Otospermophilus beecheyi*	4	Bratislava	2	0	0	0	0	0	0	2	50 (6.8-93.2)
		4	Hardjo	0	1	0	0	0	0	0	1	25 (0.6-80.6)
**Raccoon**	*Procyon lotor*	16	Autumnalis	2	0	1	0	0	0	0	3	18.75 (4-45.6)
		135	Bratislava	11	3	9	4	3	3	2	35	26.12 (18.9-34.4)
		135	Canicola	2	4	2	2	1	0	0	11	8.21 (4.2-14.2)
		16	Celledoni	1	0	0	0	0	0	0	1	6.25 (0.2-30.2)
		16	Cynopteri	1	0	0	0	0	0	0	1	6.25 (0.2-30.2)
		16	Djasiman	1	1	1	0	0	0	0	3	18.75 (4-45.6)
		135	Grippotyphosa	1	5	4	1	0	0	0	11	8.21 (4.2-14.2)
		122	Hardjo	11	5	2	4	3	0	0	25	20.66 (13.8-29)
		135	Icterohaemorrhagiae	13	9	5	5	1	2	1	36	26.87 (19.6-35.2)
		16	Mankarso	0	0	1	0	0	0	0	1	6.25 (0.2-30.2)
		135	Pomona	6	10	8	9	7	3	12	55	40.74 (32.2-49.5)
		16	Pyrogenes	1	0	0	0	0	0	0	1	6.25 (0.2-30.2)
		16	Tarassovi	0	0	1	0	0	0	0	1	6.25 (0.2-30.2)
**Red Fox**	*Vulpes vulpes*	4	Autumnalis	0	0	1	0	0	0	0	1	25 (0.6-80.6)
		4	Djasiman	0	1	0	0	0	0	0	1	25 (0.6-80.6)
		5	Icterohaemorrhagiae	1	0	0	0	0	0	0	1	20 (0.5-71.6)
		4	Mankarso	1	0	0	0	0	0	0	1	25 (0.6-80.6)
		5	Pomona	0	1	0	0	0	0	0	1	20 (0.5-71.6)
		4	Pyrogenes	1	0	0	0	0	0	0	1	25 (0.6-80.6)
**Striped Skunk**	*Mephitis mephitis*	8	Autumnalis	2	0	0	1	0	0	0	3	37.5 (8.5-75.5)
		26	Bratislava	0	1	0	0	0	0	0	1	3.85 (0.1-19.6)
		8	Djasiman	1	0	1	0	0	0	0	2	25 (3.2-65.1)
		26	Icterohaemorrhagiae	2	0	1	0	0	0	0	3	11.54 (2.4-30.2)
		8	Mankarso	1	0	0	0	0	0	0	1	12.5 (0.3-52.7)
		26	Pomona	2	0	2	0	1	0	2	7	26.92 (11.6-47.8)
**Virginia Opossum**	*Didelphis virginiana*	129	Bratislava	1	0	0	0	0	0	0	1	0.78 (0-4.2)
		75	Hardjo	7	0	0	0	0	0	0	7	9.33 (3.8-18.3)
		129	Icterohaemorrhagiae	2	0	0	0	0	0	0	2	1.55 (0.2-5.5)
		129	Pomona	0	0	0	0	0	0	1	1	0.78 (0-4.2)

Table S4: Comparison of *Leptospira* exposure and infection detected in this study centered in southern California, and by Straub and Foley (2020) centered in more northern regions of California. *Leptospira* antibody (MAT) and DNA (PCR) results are shown for our five target species.

	Common Name	Scientific Name	*Leptospira* Exposure (MAT)			*Leptospira* Infections (PCR)		
			POS	*n*	*%POS (95% CI)*	*POS*	*n*	*%POS (95% CI)*
**This Study**	Striped Skunk	*Mephitis mephitis*	6	21	28.6 (11.3-52.2)	5	33	15.2 (5.1-31.9)
	Raccoon	*Procyon lotor*	31	95	32.6 (23.4-43.0)	14	162	8.6 (4.8-14.1)
	Coyote	*Canis latrans*	14	54	25.9 (15.0-39.7)	4	108	3.7 (1.0-9.2)
	Virginia Opossum	*Didelphis virginiana*	5	97	5.2 (1.7-11.6)	1	131	0.8 (0.0-4.2)
	Fox Squirrel	*Sciurus niger*	66	109	60.6 (50.7-69.8)	0	148	0 (0.0-2.5)
**Staub and Foley, 2020**	Striped Skunk	*Mephitis mephitis*	78	206	37.9 (31.5-44.7)	40	141	28.4 (21.6-36.3)
	Raccoon	*Procyon lotor*	52	119	43.7 (35.1-52.7)	23	87	26.4 (18.3-36.6)
	Coyote	*Canis latrans*	6	20	30.0 (14.5-51.9)	2	2	100.0 (34.2-100.0)
	Virginia Opossum	*Didelphis virginiana*	2	32	6.3 (1.7-20.1)	1	6	16.7 (3.0-56.4)
	Fox Squirrel	*Sciurus niger*	15	36	41.7 (27.1-57.8)	4	31	12.9 (5.1-28.9)

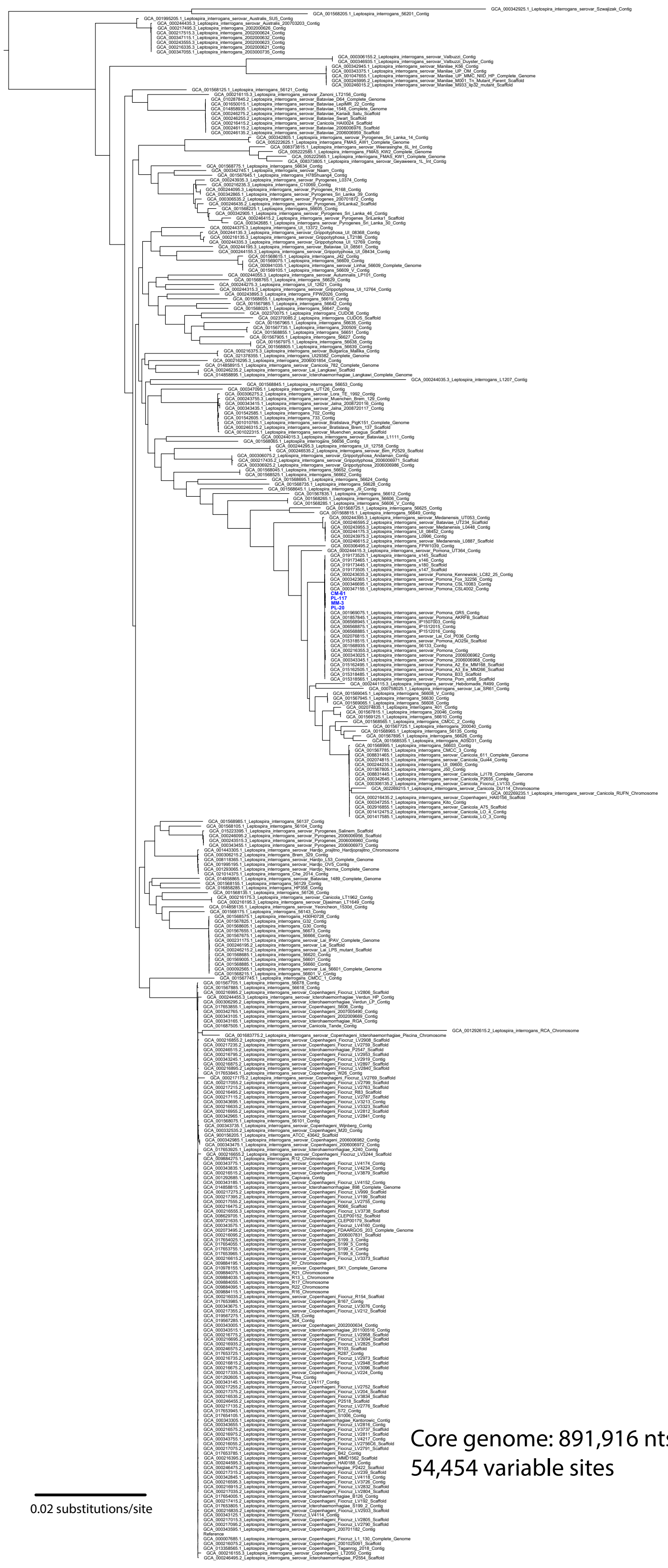

Figure S1. A maximum likelihood phylogeny of over 340 *L. interrogans* genomes together with enriched *Leptospira* genomic DNA from four PCR-positive animals in this study (highlighted with blue text) generated from one or two rounds of DNA capture and enrichment. Reads were mapped against *L. interrogans* serovar Copenhageni strain Fiocruz L1-130 (GCA_000007685.1) and single nucleotide polymorphisms (SNPs) were called with GATK. The tree was inferred by IQ-TREE using the core genome shared by all four enriched samples and the publicly available genomes, corresponding to 19.27% [891,916 of 4,627,366 nucleotides (nts)] of the reference genome.

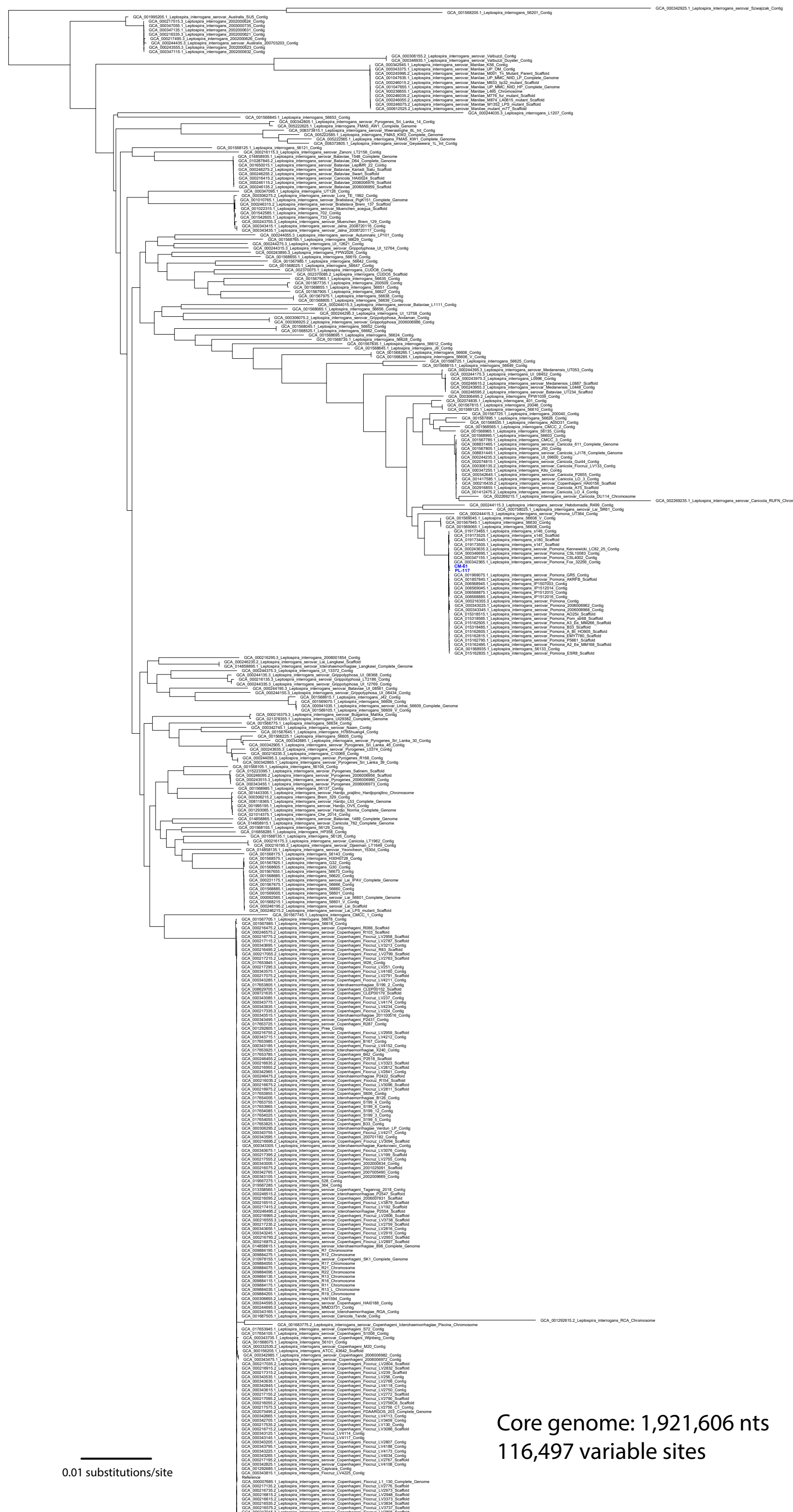

Figure S2. A maximum likelihood phylogeny of over 340 *L. interrogans* genomes together with enriched *Leptospira* genomic DNA from two PCR-positive animals in this study (highlighted with blue text) generated from two rounds of DNA capture and enrichment. Reads were mapped against *L. interrogans* serovar Copenhageni strain Fiocruz L1-130 (GCA_000007685.1) and single nucleotide polymorphisms (SNPs) were called with GATK. The tree was inferred by IQ-TREE using the core genome shared by two enriched samples and the publicly available genomes, corresponding to 41.53% [1,921,606 of 4,627,366 nucleotides (nts)] of the reference genome.

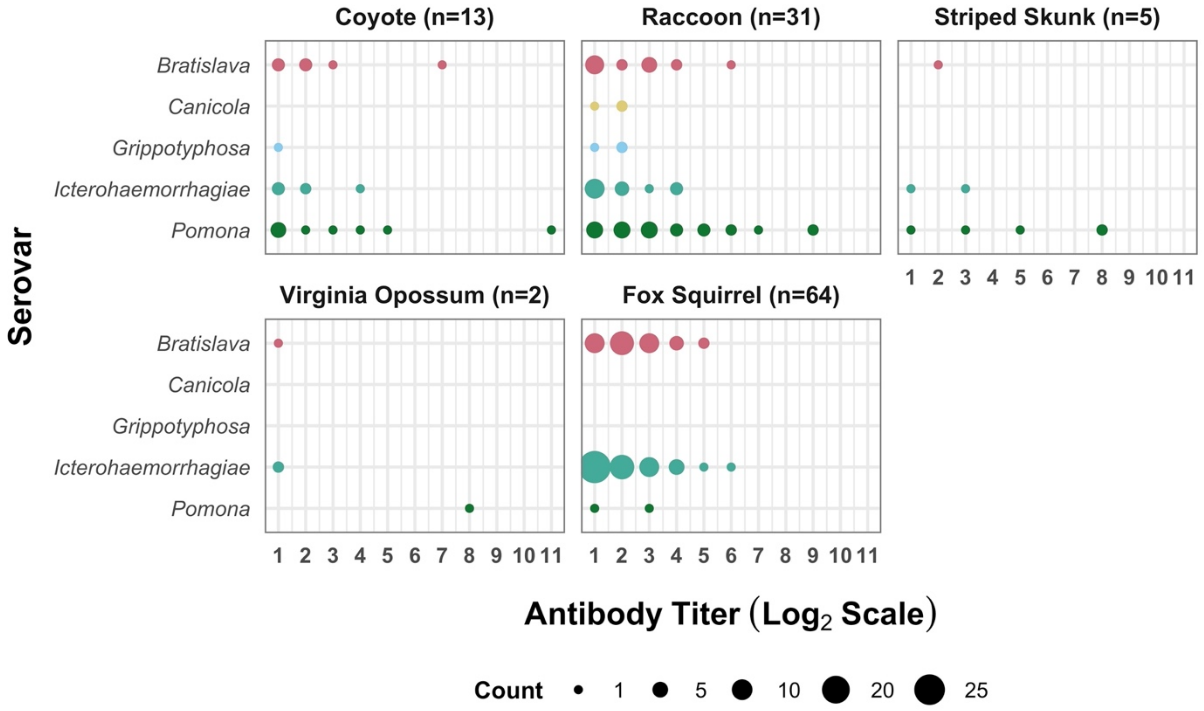

Figure S3: Samples positive for antibodies shown by host species, serovar, and titer level. Positive antibody results (MAT) for our five main serovars. Antibody titer (x-axis) is shown on a $\log_2$ scale (1:100 equivalent to 1, 1:200 equivalent to 2, 1:400 equivalent to 3, etc.).

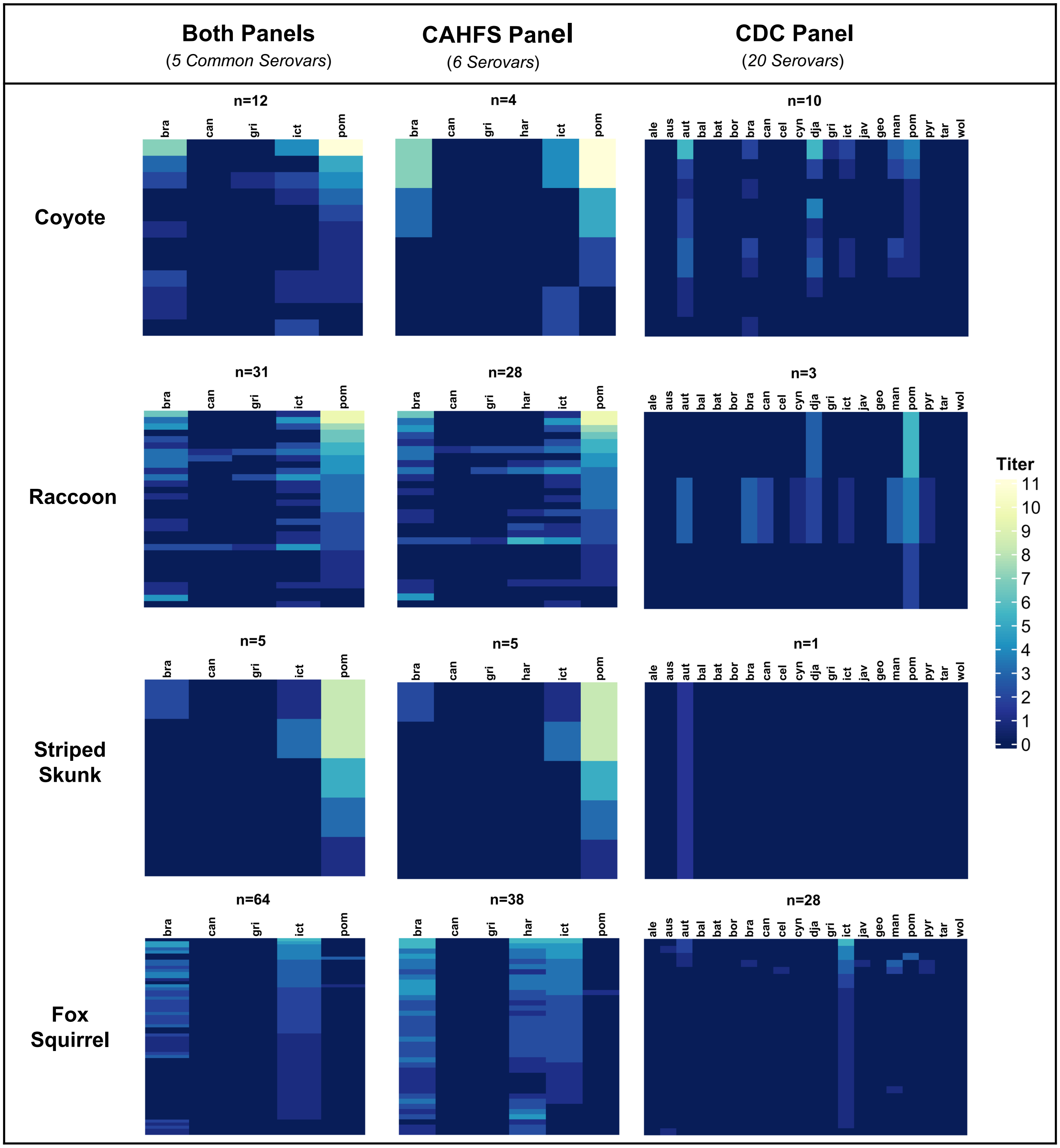

Figure S4: Serovar reactivity profiles for target species in the greater Los Angeles region, displayed by laboratory. All seropositive data are displayed for the serovars tested at both laboratories (left), CAHFS (middle) and CDC (right). Each row of colored bars represents MAT titers for an individual animal, with rows ordered by titer values for the serovar with the highest maximum titers in each species (serovar Pomona for coyotes, raccoons and skunks; serovar Icterohaemorrhagiae in fox squirrels). The colorbar legend shows antibody titer on a $\log_2$ scale (1:100 equivalent to 1, 1:200 equivalent to 2, 1:400 equivalent to 3, etc.). Serovar results shown: Alexi ('ale'), Australis ('aus'), Autumnalis ('aut'), Ballum ('bal'), Bataviae ('bat'), Borincana ('bor'), Bratislava ('bra'), Canicola ('can'), Celledoni ('cel'), Cynopteri ('cyn'), Djasiman ('dja'), Grippotyphosa (gri'), Hardjo ('har'), Icterohaemorrhagiae ('ict'), Javanica ('jav'), Georgia ('geo'), Mankarso ('man'), Pomona ('pom'), Pyrogenes ('pyr'), Tarassovi ('tar'), Wolffi ('wol').

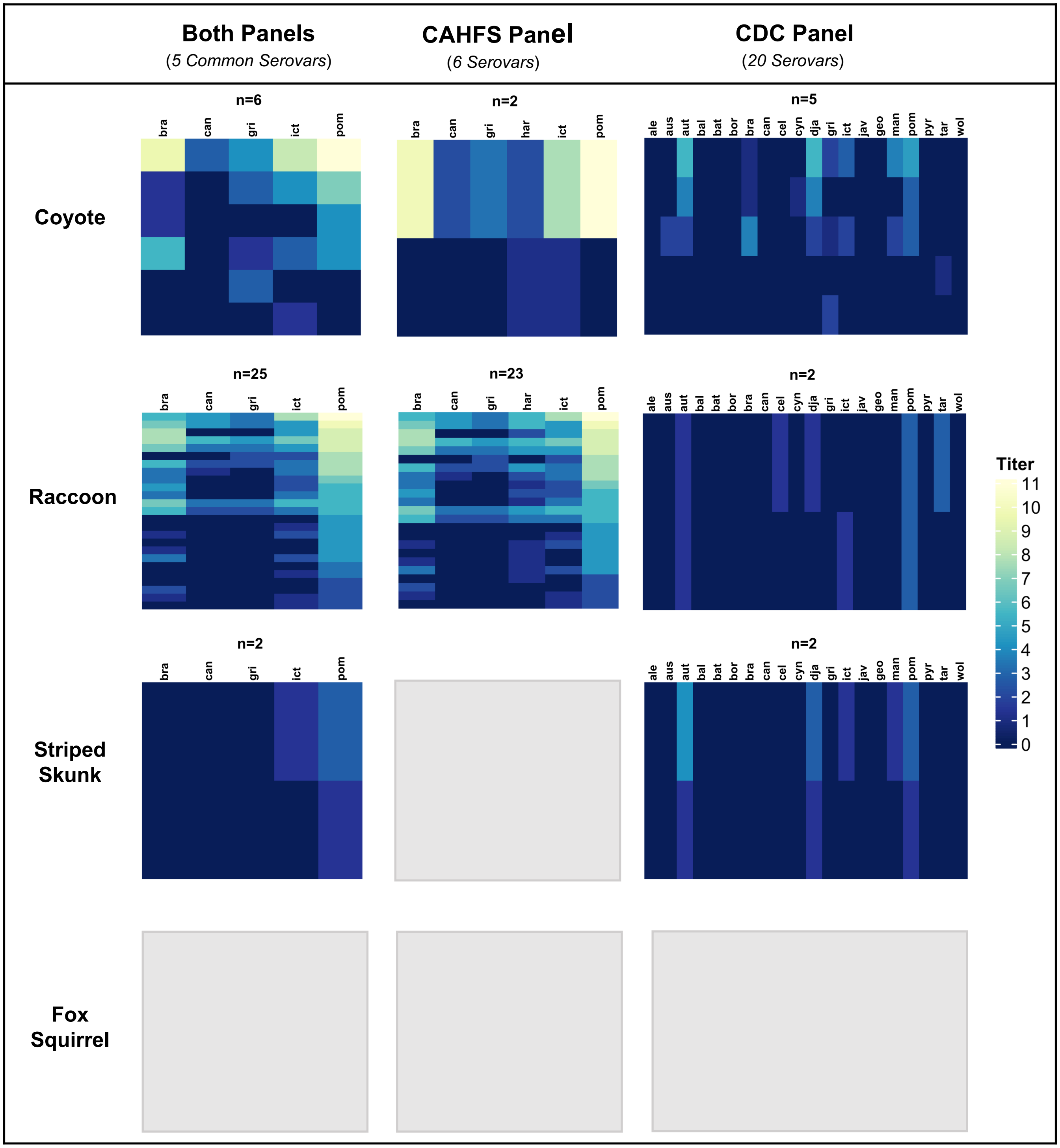

Figure S5: Serovar reactivity profiles for target species outside the greater Los Angeles region, displayed by laboratory. All seropositive data are displayed for the serovars tested at both laboratories (left), CAHFS (middle) and CDC (right). Gray boxes represent a lack of data. Each row of colored bars represents MAT titers for an individual animal, with rows ordered by titer values for the serovar with the highest maximum titers in each species (serovar Pomona for coyotes, raccoons and skunks; serovar Icterohaemorrhagiae in fox squirrels). The colorbar legend shows antibody titer on a $\log_2$ scale (1:100 equivalent to 1, 1:200 equivalent to 2, 1:400 equivalent to 3, etc.). Serovar results shown: Alexi ('ale'), Australis ('aus'), Autumnalis ('aut'), Ballum ('bal'), Bataviae ('bat'), Borincana ('bor'), Bratislava ('bra'), Canicola ('can'), Celledoni ('cel'), Cynopteri ('cyn'), Djasiman ('dja'), Grippotyphosa (gri'), Hardjo ('har'), Icterohaemorrhagiae ('ict'), Javanica ('jav'), Georgia ('geo'), Mankarso ('man'), Pomona ('pom'), Pyrogenes ('pyr'), Tarassovi ('tar'), Wolffi ('wol').

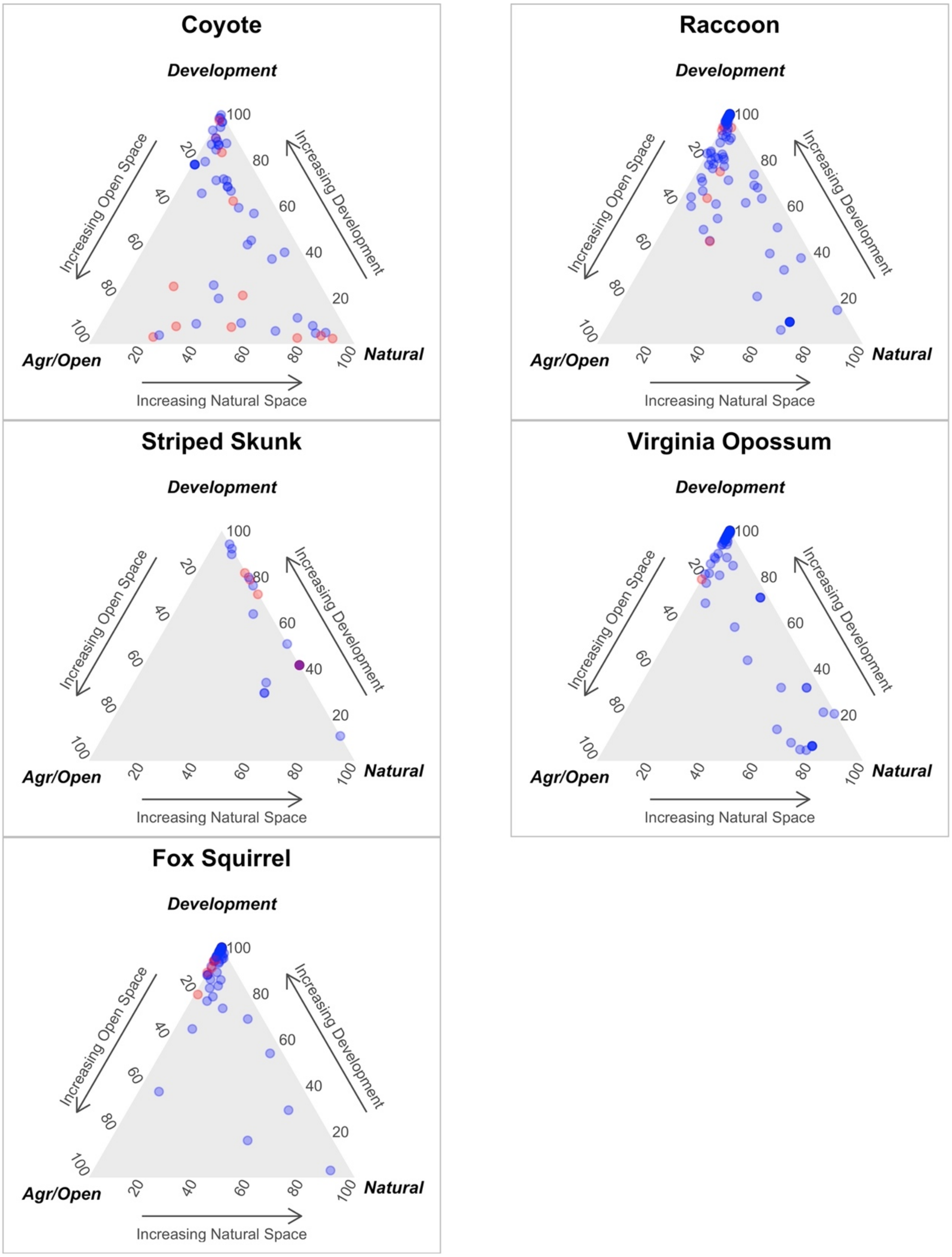

Figure S6: *Leptospira* exposure relative to land classification for target species in the greater Los Angeles region. Positive (red) and negative (blue) anti-*Leptospira* antibody (MAT) results are plotted relative to the proportion of natural, agricultural/open and developed land calculated within the home range buffer of each individual.
